# Multimodal Mass Spectrometry Identifies a Conserved Protective Epitope in *S. pyogenes* Streptolysin O

**DOI:** 10.1021/acs.analchem.4c00596

**Published:** 2024-05-03

**Authors:** Di Tang, Carlos Gueto-Tettay, Elisabeth Hjortswang, Joel Ströbaek, Simon Ekström, Lotta Happonen, Lars Malmström, Johan Malmström

**Affiliations:** † Division of Infection Medicine, Department of Clinical Sciences, Faculty of Medicine, Lund University, Klinikgatan 32, 222 42 Lund, Sweden; ‡ SciLifeLab, Integrated Structural Biology Platform, Structural Proteomics Unit Sweden, Lund University, Klinikgatan 32, 222 42 Lund, Sweden

## Abstract

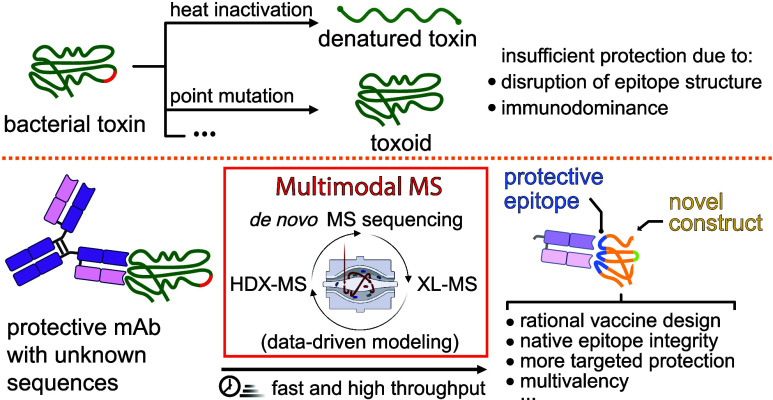

An important element of antibody-guided vaccine design is the use of neutralizing or opsonic monoclonal antibodies to define protective epitopes in their native three-dimensional conformation. Here, we demonstrate a multimodal mass spectrometry-based strategy for in-depth characterization of antigen–antibody complexes to enable the identification of protective epitopes using the cytolytic exotoxin Streptolysin O (SLO) from *Streptococcus pyogenes* as a showcase. We first discovered a monoclonal antibody with an undisclosed sequence capable of neutralizing SLO-mediated cytolysis. The amino acid sequence of both the antibody light and the heavy chain was determined using mass-spectrometry-based *de novo* sequencing, followed by chemical cross-linking mass spectrometry to generate distance constraints between the antibody fragment antigen-binding region and SLO. Subsequent integrative computational modeling revealed a discontinuous epitope located in domain 3 of SLO that was experimentally validated by hydrogen–deuterium exchange mass spectrometry and reverse engineering of the targeted epitope. The results show that the antibody inhibits SLO-mediated cytolysis by binding to a discontinuous epitope in domain 3, likely preventing oligomerization and subsequent secondary structure transitions critical for pore-formation. The epitope is highly conserved across >98% of the characterized *S. pyogenes* isolates, making it an attractive target for antibody-based therapy and vaccine design against severe streptococcal infections.

## Introduction

Antibody-guided vaccine design has emerged as a promising strategy to develop vaccines,^1^ which has shown promising results for viruses such as influenza^2^ and HIV.^3^ The approach relies on molecular information from the adaptive immune response that is harnessed to design vaccine candidates that can elicit antibody responses of high magnitude and specific activity.^4^ The recent advances in the characterization of neutralizing antibody responses, together with new protein engineering methods, have catalyzed novel opportunities to rationally design next-generation vaccines.^4^ The neutralizing/opsonic monoclonal antibodies (mAbs) are used as guides to define the protective epitopes in their three-dimensional conformation, which requires detailed information on the atomic structure of the antigen–antibody complexes.^5^ Typically, structural biology methods such as nuclear magnetic resonance (NMR) spectroscopy, X-ray crystallography, and single-particle cryo-electron microscopy (cryoEM) provide insights into the 3-D structure of antigens and the antigen–antibody complexes.^6^ These methods are, however, associated with high demands on sample quality and quantity or are limited by the molecular weight of the target antigens, impeding the identification of critically important epitopes in a high throughput manner.

Structural mass spectrometry is an emerging alternative to define epitopes of relevance for immunity. Hydrogen–deuterium exchange mass spectrometry (HDX-MS), for example, has proven useful for mapping epitopes between monoclonal antibodies and single antigens.^7,8^ Additionally, recent studies have shown that epitope mapping on small antigens can be accomplished by HDX-MS using polyclonal antibody mixtures,^9,10^ without prior knowledge of the primary structure (amino acid sequence) of the antibodies. HDX-MS can also reveal deprotected sites as a result of protein unfolding and allosteric effects,^11^ providing new information on protein dynamics in solution using a limited amount of sample. Another approach is chemical cross-linking mass spectrometry (XL-MS), which facilitates the identification of proximal structural regions at the amino acid level.^12^ Protein samples are mixed with reagents that form covalent cross-links between defined residues in solution, and upon protease digestion, the resulting peptide pairs can be identified by tandem mass spectrometry (MS/MS).^13^ The cross-linked peptide pairs generate distance constraints that can in conjunction with protein structural modeling resolve protein binding interfaces within, for example, antigen–antibody complexes.^14,15^ This method is relatively fast, does not require complicated sample preparation protocols, necessitates relatively small amounts of starting material, can be applied to most proteins, and is notably scalable to more complex mixtures of proteins.^16^ However, the primary structure information on both antigens and antibodies of interest is required.

*Streptococcus pyogenes* or group A streptococcus (GAS) is a significant human pathogen responsible for considerable morbidity and mortality worldwide.^17^ Streptolysin O (SLO), one of its major virulence factors, is a 60 kDa pore-forming toxin ubiquitously produced by GAS, and it belongs to a superfamily of pore-forming toxins as cholesterol-dependent cytolysins (CDCs). The first 69 N-terminal residues of SLO form a disordered region, followed by 3 discontinued domains (D1–D3) and a membrane-binding domain (D4) (Figure S1A).^18^ The primary role of SLO is to bind cholesterol-rich membranes and induce the cytolysis of eukaryotic cells. This depends on a multistage process that starts with SLO binding to cellular membranes, followed by oligomerization to form a prepore and a conformational shift in domain 3 to penetrate the membrane and cause cytolysis.^19^ In addition to this main biological function, SLO can also act as an immune-modulatory protein for neutrophils and impair phagocytic clearance of GAS. Subcytotoxic levels of SLO have been found to suppress neutrophil oxidative bursts, enabling GAS to resist cell killing.^20^ Immunization with SLO toxoid can counteract this inhibitory effect, although the protection in certain challenge models falls short compared to M protein immunization.^21^ Moreover, SLO-deficient GAS strains exhibit decreased virulence in mouse infection models,^22^ while antisera from SLO toxoid-immunized mice can protect naïve mice against challenges with wild-type GAS strains.^18^

SLO is highly conserved and found in more than 98% of all characterized GAS isolates across the world,^23^ positioning SLO as a promising vaccine candidate. In fact, SLO is included as one of the antigen components in at least three multivalent vaccines under investigation in preclinical and clinical trials.^24^ Current SLO-based toxoid vaccines have been developed by introducing point amino acid substitutions primarily in the membrane-binding loop of domain 4 to prevent cytolysis during immunization.^18,25^ However, the rational design of SLO-based vaccines against GAS has not yet been demonstrated. Unlike the M protein, there are no reports to date of protective monoclonal antibodies with characterized primary structures targeting SLO, which impede the identification of the protective epitope(s). In contrast, several cytolysis-inhibiting mAbs have been well characterized for pneumolysin, a homologous CDC secreted by *Streptococcus pneumoniae;* however, the epitope mapping was mainly conducted using overlapping fragments and peptides.^26^

In this study, we propose a novel multimodal mass spectrometry strategy and sequenced a neutralizing monoclonal antibody to determine the protective epitope by investigating the SLO-antibody complex in its native state. The epitope is located in domain 3 and is conserved across >98% of the GAS genomes. The findings also showcase multimodal mass spectrometry as a versatile and promising strategy for identifying protective epitopes within bacterial antigens.

## Experimental Section

### Protein Production and Purification

Wild-type SLO protein was either commercially obtained (Sigma-Aldrich) or produced and purified in-house as previously described^27^ (Supporting Information Methods). The purity and sequence integrity of the proteins were verified using SDS-PAGE (Bio-Rad) and bottom-up mass spectrometry (BU-MS).

### Hemolysis Inhibition Assay

The murine-neutralizing antibody (nAb) was purchased from Abcam (AB23501, Lot No: GR3430639). The nAb binding specificity against SLO or the derivative d3_m construct was determined by ELISA. In the SLO-mediated hemolysis inhibition assay, a diluted suspension of sheep erythrocytes was treated with active SLO and antibody samples or a PBS buffer control, with the extent of released hemoglobin indicating the antibody/fragments hemolysis inhibition efficacy (Supporting Information Methods).

### *De Novo* Sequencing, Assembling, and Modeling of the Fragment Antigen-Binding Domain

*De novo* MS sequencing and assembly of the nAb were conducted through BU-MS, utilizing four proteases to generate overlapping digested peptides for LC–MS analysis. A novel approach considering cumulative fragment-ion evidence was applied to enhance *de novo* peptide sequencing and follow-up nAb primary structure assembly. The fragment antigen-binding (Fab) domain of the nAb was predicted using AlphaFold-Multimer (v2.3.1) (Supporting Information Methods).

### XL-MS Experiment, DisVis, and HADDOCK Analysis

The in-solution XL-MS experiment was performed on the SLO-nAb complex using two cross-linkers with different spacer arm lengths, following a methodology elaborated in prior studies.^15,28^ The collected distance constraint information was carefully analyzed, interrogated, and then applied in a distance-information-driven docking procedure using HADDOCK 2.4 suites as explained in Supporting Information Methods.

### HDX-MS Experiment and Data Analysis

The experiment was conducted on both the unbound (apo) SLO and the nAb-bound SLO to identify the Fab-recognized regions as previously demonstrated.^7^ Deuteros 2.0 was employed for the determination and visualization of the protected regions indicative of the nAb-targeted epitopes (Supporting Information Methods).

### Conservation Analysis and Design of d3_m Construct

To investigate the conservation of the targeted epitope, a curated BLAST database was established, compiling 2216 high-quality GAS genomes. Utilizing ProteinMPNN and AlphaFold2, a novel d3_m construct was designed by integrating two discontinuous regions, with the process detailed in the Supporting Information Methods.

## Results and Discussion

### *De Novo* Sequencing and Modeling of a Neutralizing Monoclonal Antibody

**Figure 1:**
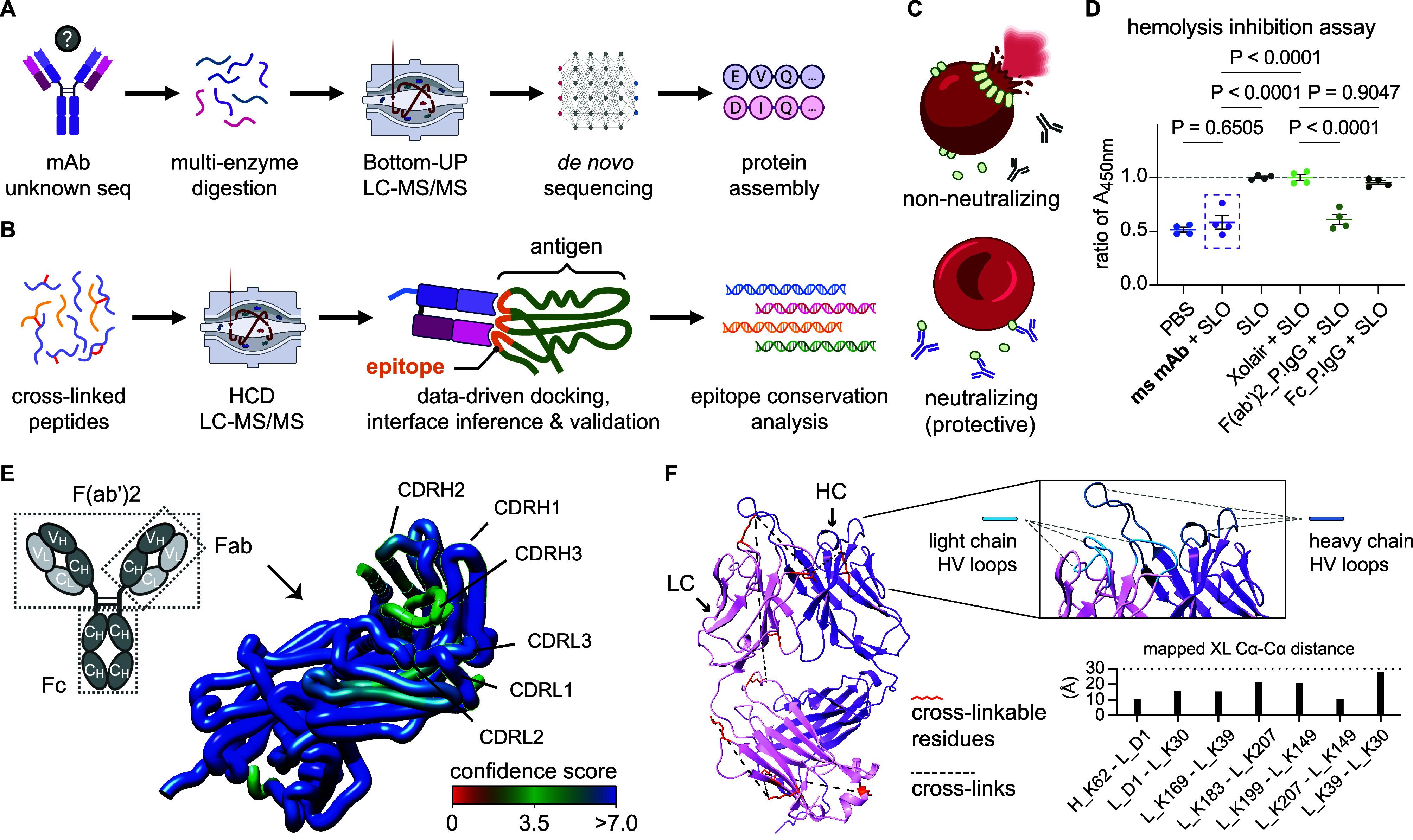
Illustration of the multimodal MS workflow outlining (A) protein sequencing of the monoclonal antibody (mAb) using multienzyme digestion, BU-MS, *de novo* sequencing, and protein sequence assembly, (B) followed by chemical cross-linking mass spectrometry to generate distance constraints for pairwise antigen–antibody complex modeling, experimental validation and assessment of epitope conservation. (C) Schematic representation of the inhibition assay used to define neutralizing antibodies. (D) Hemolysis inhibition was studied by coincubating different antibody samples with wild-type active SLO, then added to diluted sheep erythrocyte suspension. The antibody-mediated inhibition of hemolysis was quantified by measuring the amount of released hemoglobin in the supernatant (ms mAb = a murine IgG1 mAb, Xolair = a murine anti-IgE IgG1 mAb, P.IgG = isolated human IgGs from convalescent plasma of a single donor). The absorbance at 450 nm (*A*_450nm_) is normalized against the positive control (SLO). P values from the ANOVA group analysis are indicated. (E) Left, a schematic illustration of an IgG1 antibody, illustrating two heterodimeric chains comprised of both variable (V_L_ and V_H_) and constant regions (C_L_ and C_H_). Right, a tertiary model generated by AlphaFold-Multimer of the *de novo* sequenced Fab domain, with the confidence score for each residue shown by the color gradient. (F) To validate the model, XL-MS was applied to identify inter/intralinks in the Fab. Left, cross-links are displayed as pseudo bonds connecting reactive residues within the predicted structure. Light chain (LC) and heavy chain (HC) are distinguished by color, and the proABC-2-predicted hypervariable (HV) loop regions are highlighted by light or dark blue, respectively. The bar plot at the bottom of the right shows a summary of all identified cross-links mapped on the Fab structure, with the *y*-axis representing the Cα–Cα distance measurement.

In our proposed multimodal mass spectrometry workflow, epitopes targeted by a monoclonal antibody (mAb) are identified by first using *de novo* mass spectrometry sequencing to determine the primary structure of mAb (Figure 1A) and then XL-MS to generate distance constraints within the antigen–antibody complex. In the following phase, the epitopes are defined by an information-driven docking protocol to predict the most important residues in the binding interface between the fragment antigen-binding (Fab) region and the antigen that can be experimentally validated by HDX-MS and reverse engineering of the epitope (Figure 1B).

Antibody protection can be divided into neutralization and/or opsonization. In this study, we defined antibody protection by the degree of neutralization of SLO-mediated cytolysis (Figure 1C). As a starting point, we searched readily available anti-SLO mAbs from public and commercial sources that could bind to SLO with high specificity, leading to neutralization of cytolysis. Using an indirect ELISA, we identified a murine mAb that bound specifically to SLO (Figure S1B). Neutralization was further assessed by incubating different antibody samples with cytolytic SLO, followed by the addition of a diluted sheep erythrocyte suspension. Inhibition of hemolysis was quantified by measuring the reduction of the released hemoglobin in the supernatant. The neutralizing mAb (nAb) completely inhibited the SLO-mediated hemolysis compared to the incubation with the unrelated murine IgG1 Xolair (Figure 1D). To further investigate whether neutralizing antibodies occur after bacterial infection, we isolated IgGs from the plasma of a single donor recovering from a recent GAS infection. By separately comparing the neutralizing effect of the F(ab’)2- and Fc-fragments, we could demonstrate that neutralizing antibodies can arise after a GAS infection and that neutralization is mediated by the Fab domain alone (Figure 1D,E, top-left).

To obtain the full amino acid sequence of the nAb, we digested the antibody using four different proteases with different cleavage specificities and used a combined *de novo* sequencing strategy based on three different search engines to sequence proteolytically overlapping nAb peptide fragments. The overlapping peptide fragments were assembled into a high-confidence full-length sequence including the complementary determining regions (CDRs) involved in the antigen binding for both the light and heavy chains, followed by modeling of the tertiary structure of the Fab domain (Figure 1E, right). To validate the model, we used AlphaFold-Multimer to predict the Fab structure (ipTM + pTM = 0.848; interchain pDockQ = 0.688) followed by XL-MS to generate distance constraints within and between the nAb heavy chain (HC) and the light chain (LC) (Table S1). The identified interlinks and intralinks conformed to the modeled Fab structure without violating the Cα–Cα upper distance limit of 30 Å (Figure 1F, left and bottom-right). Furthermore, prediction of the antibody residues and type of interactions involved in the intermolecular interaction with antigens were determined by proABC-2. This analysis pinpointed residues 26–32, 50–52, and 91–96 in the light chain as hypervariable (HV) contact loop regions and the residues 26–31, 52–57, and 100–114 in the heavy chain as HV loop regions (Figure 1F, top-right). Collectively, the identified distance constraints show that the primary structure together with the modeled Fab structure is of sufficient quality to infer the epitope through XL-MS and information-driven docking.

### Proximation of Binding Sites by XL-MS and Epitope Inference from Interaction Analysis

**Figure 2:**
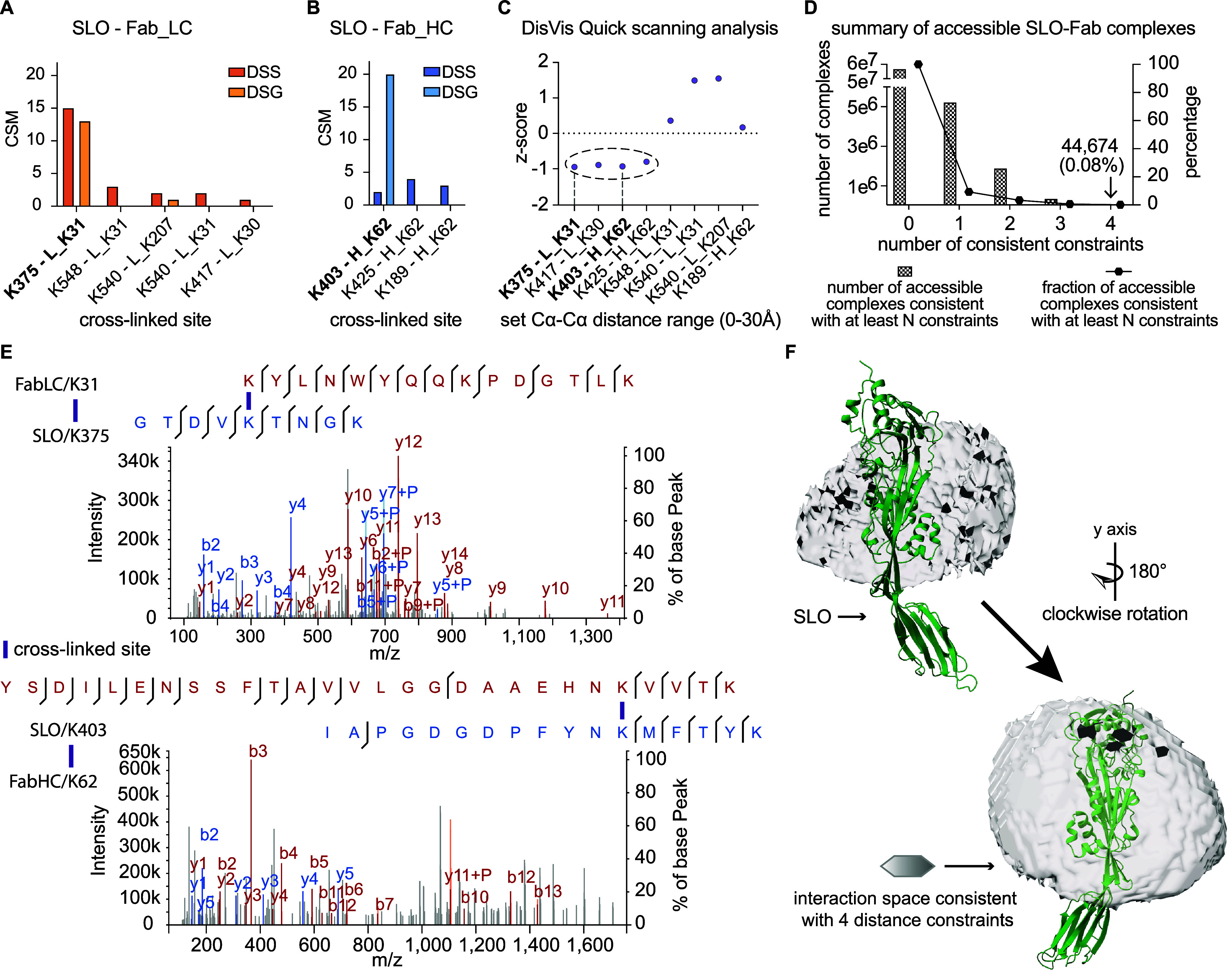
(A,B) The summary of cross-linked peptide spectrum match (CSM) for the identified DSS and DSG interprotein cross-linked peptide pairs between SLO-Fab light chain and SLO-Fab heavy chain. K, lysine; L, light chain; H, heavy chain. (C) DisVis Quick scanning analysis was used to filter experimentally derived interprotein distance constraints. DSS XLs are plotted on the *x*-axis with the calculated *z*-score values on the *y*-axis. The dotted circle indicates the group of four distance constraints with lower *z*-scores that were selected for downstream analysis. (D) Histogram showing the reduction of accessible complexes according to the number of distance constraints to apply. The remaining 44,674 complexes were consistent with all four distance constraints. (E) Representative MS/MS spectra of the two predominant cross-linked peptide pairs are presented with annotated fragmented ions. The top and lower panels represent the FabLC/K31-SLO/K375 and the SLO/K403-FabHC/K62 cross-links, respectively. (F) Accessible interaction space formed by the filtered 44,674 complexes, shown as a cloud-like shell centered around domain 3 in SLO.

To identify the epitope targeted by the nAb, we incubated SLO with the nAb at a 1:1 molar ratio followed by a cross-linking reaction using disuccinimidyl glutarate (DSG) or disuccinimidyl suberate (DSS). Subsequent mass spectrometry analysis identified five cross-linked peptide pairs between SLO-Fab/LC and three pairs between SLO-Fab/HC (Table S2). The DSG and DSS cross-linkers have different spacer arms, resulting in partially overlapping peptide pairs. Three of the cross-linked peptide pairs were identified by multiple independent cross-linked peptide spectrum match (CSM) for both DSS and DSG (Figure 2A,B).

**Figure 3:**
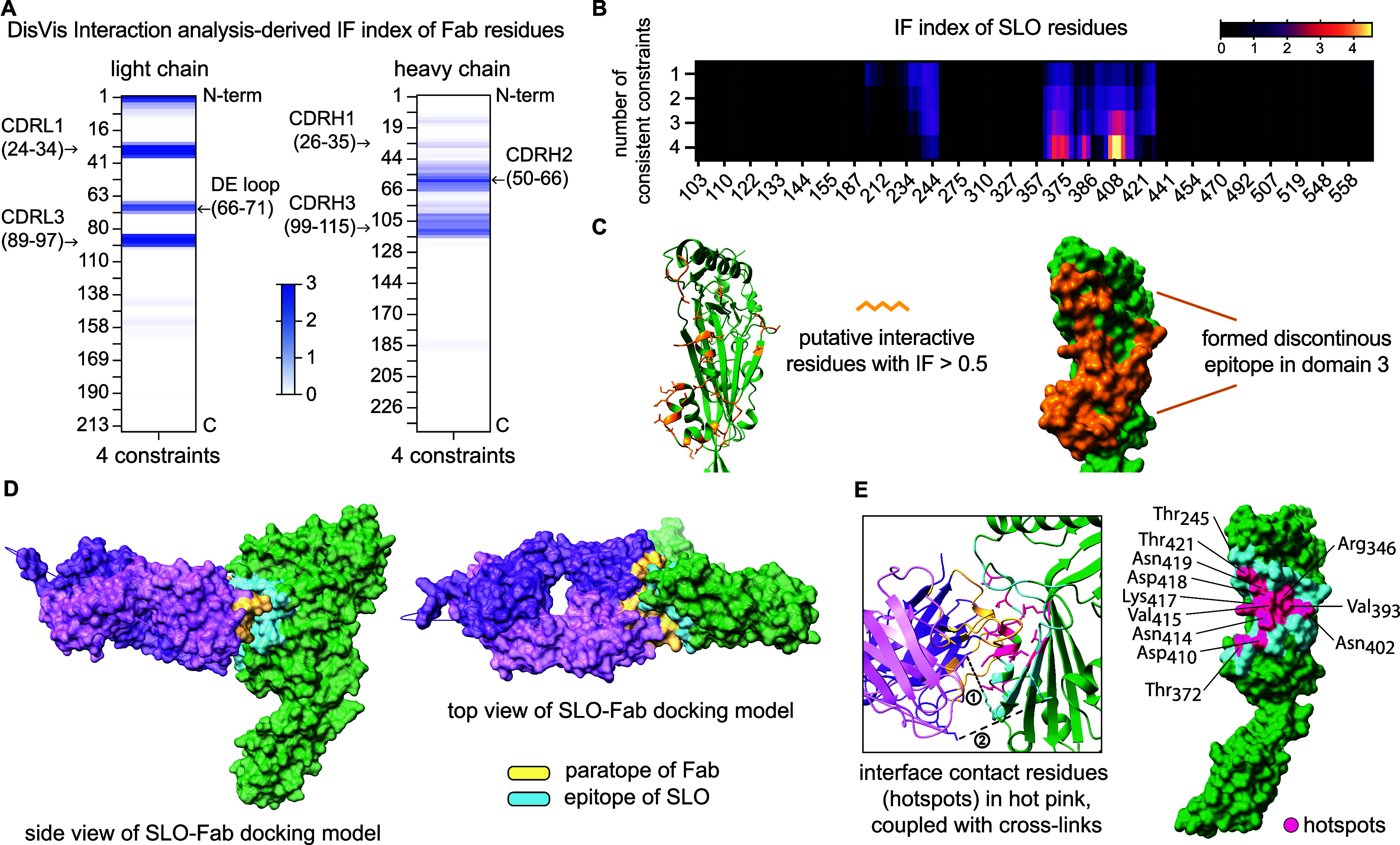
(A). Heatmaps displaying the interaction fraction (IF) index of each accessible residue from the light and the heavy chain separately, with a color gradient indicating an index from 0 to 3. (B) Heatmap showing the IF index of all SLO surface-accessible residues based on the number of distance constraints applied in analysis, with an IF value ranging from 0 to 4.6. (C) Left, display of most likely interactive residues (with an IF > 0.5) with side chains shown as sticks on the tertiary structure, colored in light orange. Right, formed conformational epitope holding these putative interactive residues, shown in the surface presentation on the SLO structure, colored in dark orange. (D) Top-ranked model generated by HADDOCK 2.4 antigen–antibody docking protocol with (i) a loose XL-MS derived epitope definition, (ii) predicted Fab paratope as hypervariable (HV) loop contacts, and (iii) the two most frequently observed distance constraints as inputs. The PRODIGY-predicted epitope and paratope from the top modeled pairwise SLO-Fab complex are color-coded correspondingly. (E) Interface contact residues, highlighted in hot pink, were identified and classified in the indicated SLO-Fab structure, with the side chains displayed in sticks and cross-links in proximity. ①: SLO-FabLC; ②: SLO-FabHC. The residues, defined by the hotspots to represent those predicted to contribute most significantly to the intermolecular interaction, are labeled with residue names and numbers, and annotated on the SLO structure in surface presentation.

Experimentally derived distance constraints that define or infer the binding interface are of importance to guide the modeling of antibody–antigen complexes and to improve the accuracy of the deep learning algorithms.^29^ To select the most informative distance constraints, DisVis Quick scanning analysis was applied to all identified interprotein cross-links (XLs). Four of the XL distance constraints displayed a negative *z*-score, an indicator of relevance, and were selected for further analysis (Figure 2C). The two XLs with the highest number of CSM were included among these four selected distance constraints. Next, these four filtered XLs were used to guide the DisVis Interaction analysis, to identify the putative residues, and to infer the binding interface. By setting SLO as the fixed molecule and Fab as the scanning molecule without using any XL distance constraints, nearly six million pairwise conformations were generated as an initial interaction space. This large conformational space was produced by allowing the Fab molecule to freely translate and rotate relative to the fixed SLO molecule. All surface-accessible residues on both interacting partners, with a relevant solvent accessibility (RSA) value greater than 40%, were considered interactive residue candidates (Table S3). This approach was aimed to provide a complete representation of all possible interaction scenarios between SLO and the Fab fragment (Figure S1C). The stepwise inclusion of the selected four most informative cross-links (Table S4) filters the interaction space by removing SLO-Fab conformations that violated the applied distance constraints, reducing the number of conformations to a final set of 44,674 (0.08% of initial space) when including all four XLs (Figure 2D). Representative MS/MS spectra for the two most frequently observed cross-linked peptide pairs are presented (Figure 2E). The final interaction space, produced from all possible SLO-Fab complexes using the filtered 44,674 conformations, revealed that the epitope resides within domain 3 of SLO (Figure 2F). Finally, we used the interaction fraction (IF) index to predict the residues of importance for the antigen–antibody binding interface (Figure S1D). Calculating the involvement of each surface residue in forming an interface within SLO-Fab complexes, consistent with derived distance constraints, enables the IF value to accurately reflect its likelihood of contributing to intermolecular interactions. On the Fab side, the most important residues with an IF index higher than 0.5 were located in the CDRs for both the heavy and the light chains (Figure 3A). Reciprocally, the putative interface residues in SLO were located in two distinct discontinuous regions between amino acids 187–244 and 357–440 with the latter region being the more significant one based on the IF index profile (Figure 3B). Highlighting this region on the crystal structure of SLO (PDB: 4HSC) shows that the epitope is discontinuous and located within domain 3, and composed of 3 alpha-helices and 2 β-sheets (Figure 3C).

### Interface Prediction via Distance-Information-Driven Modeling of SLO-Fab Complex

The DisVis analysis, the IF index, along with acquired XL distance constraints enabled the prediction of a loosely defined epitope located in domain 3 of SLO. To further determine the interaction site, we used the HADDOCK 2.4 antigen–antibody docking protocol. This protocol uses the interface information from DisVis to generate more accurate starting conformations for iterative docking and the refinement of pairwise protein complexes. We tested different information inputs such as (i) no epitope information, (ii) loose epitope definition, (iii) loose epitope definition + XL distance constraints, to perform the docking process separately and found out that the last input option, DisVis-derived loose epitope combined with the two most frequently observed XL distance constraints (SLO/K375-FabLC/K31 and SLO/K403-FabHC/K62) as the center of mass, resulted in the most significant cluster comprising 240 of the 400 generated models (Table S5). The top-ranked SLO-Fab pairwise complex model from this cluster holds a combined HADDOCK score of −145, where normally a combined score less than −120 is considered a confident score.^30−32^ The selected top-ranked SLO-Fab model is depicted from two perspectives (Figure 3D), highlighting the paratope and epitope composed of interface residues as predicted by PRODIGY. Next, the SpotOn algorithm, which predicts possible side chain interaction between pairs of residues within the pairwise protein complex, was applied to this top modeled SLO-Fab structure to identify the most important interface contact residues, referred to as hotspots (Figure 3E, left; Table S6) coupled with approximate cross-links. This analysis indicates, in particular, that residues Thr_245_, Arg_346_, Thr_372_, Val_393_, Asn_402_, Asp_410_, Asn_414_, Val_415_, Lys_417_, Asp_418_, Asn_419_, and Thr_421_ in SLO are significantly involved in forming the binding interface between SLO and the nAb Fab domain (Figure 3E, right).

### Applying HDX-MS for Validation of Epitopes Derived by XL-MS and Docking Approaches

**Figure 4:**
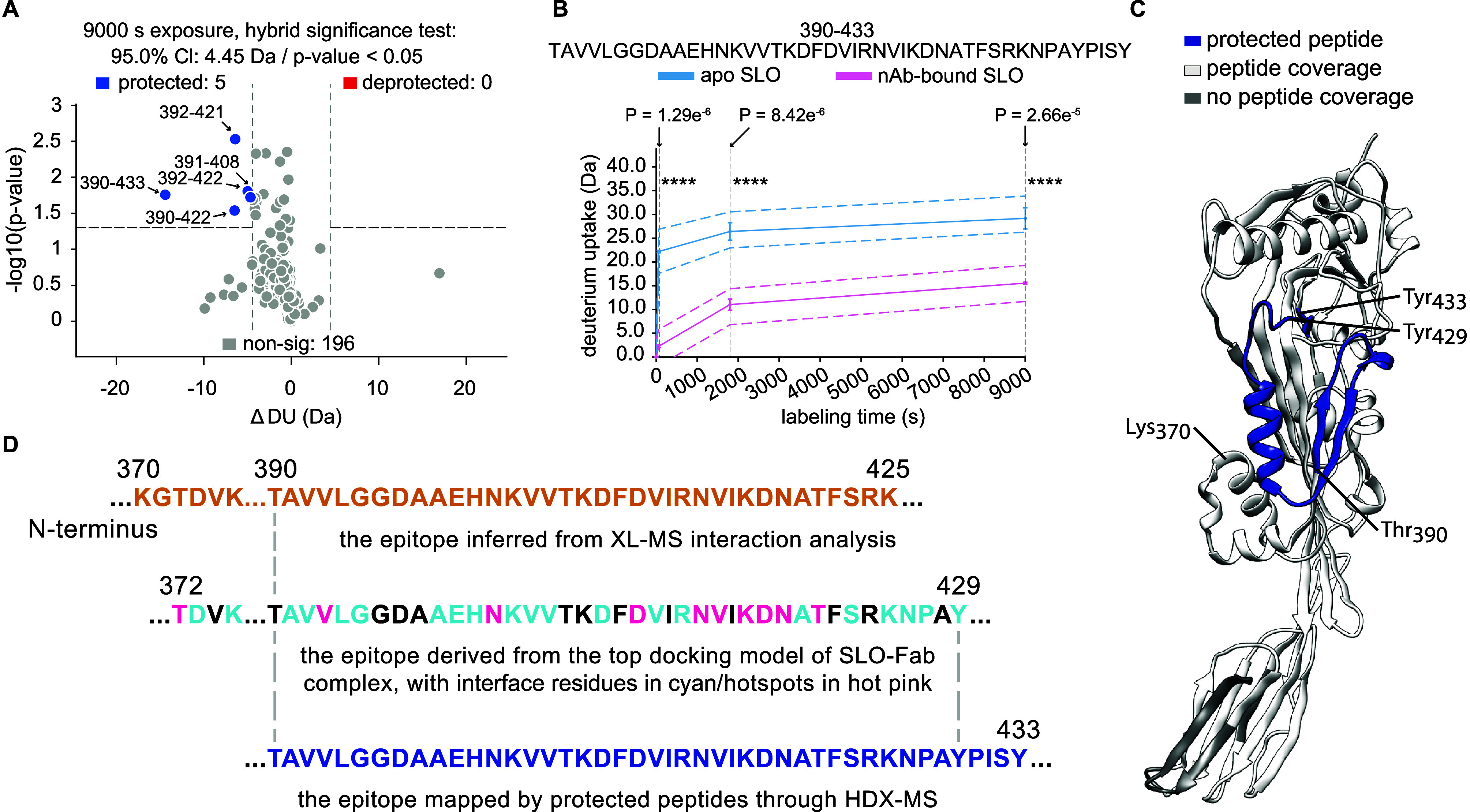
(A) Volcano plot showing significantly protected and deprotected peptides, with thresholds set at |ΔDU| > 4.45 Da and a *p*-value <0.05. (B) Kinetic plots displaying one representative protected peptide of SLO without/with nAb binding across 0, 60, 1800, and 9000 s deuteration intervals. Thresholds were globally calculated with “ns” as nonsignificant and “****” as ≥99.9% significance. (C) 3-D structure of SLO revealing the protected peptides after 9000 s labeling. Here, residues of the protected peptides are colored in blue, while residues not detected in the HDX-MS analysis are depicted in dark gray. (D) Alignment and comparison of the three epitopes on SLO derived from (i) XL-MS interaction analysis, (ii) information-driven docking, and (iii) HDX-MS, highlighted in colors accordingly, with residue number labeled on top of the sequences.

To validate the results from the distance-information-driven docking, we used HDX-MS to investigate changes in the deuterium uptake of SLO residues when bound to the nAb and to further investigate any alterations in SLO protein dynamics upon nAb binding. The HDX-MS experiments generated high-confidence annotation of 202 common peptides corresponding to a sequence coverage of 95.7% with a high degree of overlap (Figure S1E). The changes in deuterium uptake (ΔDU) over four deuteration intervals (0, 60, 1800, and 9000 s) for each identified peptide were aggregated and shown in a butterfly plot (Figure S1F). Overall, the changes in deuterium uptake between apo (unbound) SLO and nAb-bound SLO states were slight, except for the peptides starting from Thr_390_ to Phe_409_. The hybrid significance test identified the SLO peptides spanning 390–422, 390–433, 391–408, 392–421, and 392–422 to be significantly protected during the interaction with nAb (Figure 4A). The kinetic plot illustrates deuterium uptake changes of one representative peptide (390–433) across all deuteration intervals, with the level of significance indicating protection due to nAb binding (Figure 4B). The protected peptides were all found in domain 3 of SLO (Figure 4C), and near-perfectly overlapped with the epitopes predicted from the XL-MS interaction analysis and the SLO-Fab complex docking model (Figure 4D). HDX-MS results can also be used to exclude less representative XLs. In our case, HDX-MS data did not support the interaction sites directed by several distance constraints (SLO/K548-FabLC/K31, SLO/K540-FabLC/K31, SLO/K540-FabLC/K207 and SLO/K189-FabHC/K62) identified in previous experiments, which were considered to be weak or transient interactions captured by in-solution XL-MS. Future comparison with epitopes on SLO, mapped by isolated IgGs, could yield insights of higher relevance for immunity and in vivo protection. Importantly, the HDX-MS results further show that SLO does not undergo major conformational change upon nAb binding. This emerging concept of integrating several MS-based techniques extends new opportunities to enrich structural information and protein dynamics for other generic protein–protein complexes, which require considerable effort to crystallize, or which do not crystallize at all.

### Assessment of Epitope Identification Confidence Using a Reverse-Engineered Construct

**Figure 5:**
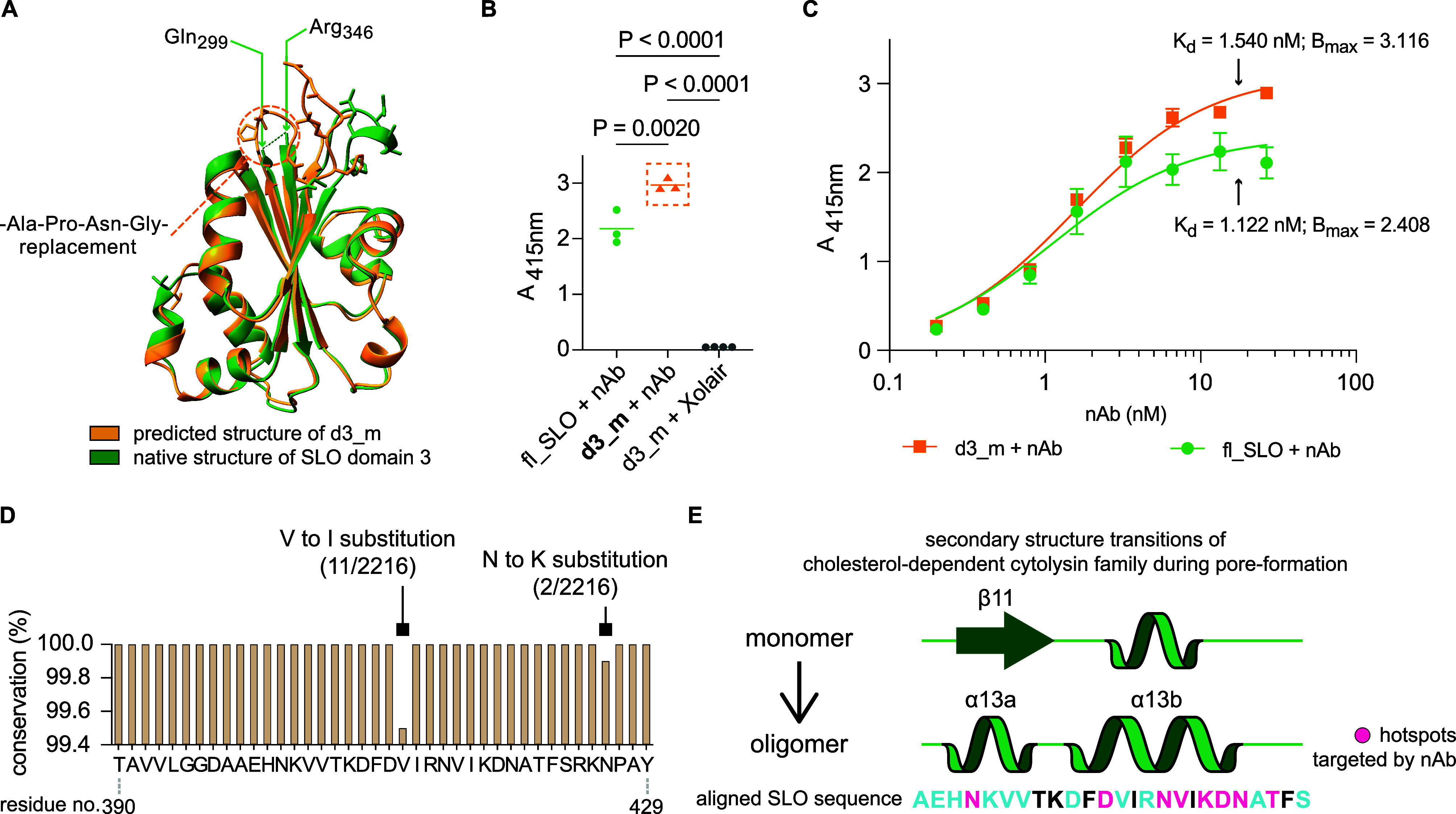
A) Structure superimposition and alignment were performed on the predicted structure of the novel construct d3_m and the corresponding native domain 3 extracted from the crystal structure of SLO (PDB ID: 4HSC). The visualization of the side chain represents the conformationally dissimilar loop regions. “-APNG-” replacement linker and two breakage points of native domain 3 are annotated. (B,C) ELISA was implemented to assess the nAb specificity against the d3_m construct by one-way ANOVA group analysis, and also to estimate the binding kinetics via the equilibrium dissociation constant (*K*_d_) and maximum specific binding (*B*_max_), derived from the nonlinear regression analysis. Adjusted *P* values are added accordingly. fl_SLO: full-length SLO protein. (D) Conservation level of the protective epitope across 2216 available GAS genomes. The bar indicates the degree of conservation for the residues that constitute the determined epitope. (E) Streptolysin O, similar to other cholesterol-dependent-cytolysins, undergoes conformational rearrangement, particularly in the secondary structures of domain 3. The nAb Fab-recognized epitope colored as in Figure 4D overlaps quite a portion of these structurally critical motifs shown as green cartoons representing the α helix and β-sheet.

In the final experiment, we redesigned and produced a new protein construct, termed d3_m, to verify that the identified discontinuous epitope was sufficient for nAb binding. Here, we used in-silico stabilization analysis to merge the two noncontinuous regions of domain 3 that were identified by XL-MS and HDX-MS by replacing the stretched region from Gln_299_–Gly_345_ with a shorter linker sequence of four amino acids (Ala–Pro–Asn–Gly) (Figure S2A). The entire d3_m construct is 128 amino acids long and is 76.3% shorter than the full-length SLO. Modeling of the d3_m construct revealed a high degree of structural similarity to the native domain 3. The AlphaFold2-predicted model showed a root-mean-square-deviation (RMSD) of 0.714 Å from the original structure, indicative of a close alignment (Figure 5A). To determine whether the nAb could recognize this newly modified construct, we expressed and purified d3_m from *E. coli* and evaluated the binding specificity using indirect ELISA. The nAb binds specifically to d3_m in an equal manner compared to the full-length SLO, while an unrelated IgG1 (Xolair) showed no binding specificity (Figure 5B). Furthermore, the equilibrium dissociation constant as *K*_d_ determined by nonlinear regression analysis indicates that the nAb binds marginally stronger to intact SLO compared with the binding to d3_m (Figure 5C). A higher *B*_max_ (maximum specific binding) value of d3_m, on the other hand, demonstrated better epitope accessibility for the nAb on this novel construct.

### Conservation Analysis of the Determined Epitope and Protective Mechanism Speculation

For the final analysis, we first mined streptococcal genomes available in the public domain to investigate if this epitope is conserved in GAS. Among the available 2216 GAS and 154 *Streptococcus dysgalactiae* genomes, 98% of the GAS and 56% of the *S. dysgalactiae* strains carried the SLO gene using a >98% sequence similarity compared to the reference SLO protein with UniProtID: P0DF96. These results indicate that rationally designed vaccines directed against this epitope region could potentially confer cross-reactive protection against both streptococcal species. Next, we assessed the level of conservation for the epitope by querying the epitope sequence in 2216 publicly available GAS genomes. Within all genomes that carried SLO, the epitope was nearly 100% conserved apart for 0.6% of the SLO sequences having a semiconserved amino acid point substitution from valine to isoleucine, and/or asparagine to lysine (Figure 5D). Like other CDC family members, Streptolysin O forms a pore on a cholesterol-rich membrane through a multistage process that includes initial binding to the cellular surface followed by oligomerization. During this process, domain 3 undergoes a significant conformational shift including (i) the induction of refolding where the hotspot Thr_245_ is found in a previously reported pivotal loop region,^19^ and (ii) the transformation of the D3 central β sheet 11 to α helix 13 where the hotspot Asn_402_ is located (Figure 5E). These secondary structure shifts trigger oligomerization, prepore formation, and final assembly into a pore. Both the XL-MS and HDX-MS results show that the nAb binds precisely to this critical region responsible for oligomerization, hence possibly preventing the secondary structure transitions required for the subsequent pore-formation. Understanding the protection exerted by the nAb demands further investigation of its mode of action and binding avidity. An interesting future prospect would be to examine and compare the epitopes of other SLO-homologous cytolysins, like pneumolysin,^26^ which are recognized by different functional mAbs.

## Conclusions

In this study, we demonstrate the versatility of protein mass spectrometry by (i) *de novo* sequencing a neutralizing monoclonal antibody that inhibits SLO-mediated cytolysis and (ii) using the orthogonal capabilities of XL-MS and HDX-MS combined with integrative computational modeling to identify a protective epitope within the antigen–antibody complex in its native state. Here, *de novo* MS-based sequencing provides data critical for distance constraint determination in XL-MS and the following information-driven docking. Leveraging both XL-MS and HDX-MS enabled a more comprehensive characterization of the epitope. XL-MS is limited by the absence of accessible cross-linkable residues or unknown primary structures that can be overcome by HDX-MS. Reciprocally, XL-MS provides informative distance constraints to approximate binding interfaces, including those involving hydrophobic residues, which is challenging to obtain by HDX-MS partly due to the peptide-level resolution associated with the setup used here. Implementing several complementary methods can considerably enrich the structural information compared to any stand-alone method as previously shown.^33,34^ This study highlights the potential of a multimodal MS strategy for identifying epitopes, investigating antibody binding modes, and analyzing protein–protein interactions across various bacterial antigens. Employing this integrated approach expands the landscape of protective epitopes that can serve as a starting point for the prospective development of both passive and active immunity intervention strategies, including antibody reengineering and vaccine design. However, a deeper understanding of the protective mechanisms requires a more systematic assessment of epitopes recognized by a broader range of functional antibodies. Such insights could catalyze future antibody-guided vaccine design against bacterial pathogens and contribute to the ongoing efforts aimed at developing a next-generation vaccine against GAS.

## Supporting Information Available

SLO domain annotation; mAb binding specificity; interaction space consistent with 1–4 distance constraints; interaction fraction (IF) value of residues; common peptide redundancy and sum of deuterium uptake in HDX-MS; redesign process of the novel d3_m construct based on domain 3 of SLO; summary of cross-linked peptide pairs; definition of surface-accessible residues; distance constraints applied in interaction analysis; summary of docking clusters; list of most important interface residues; and detailed information on materials, experiments, and methods (PDF)

## References

[ref1] Pantaleo G.; Correia B.; Fenwick C.; Joo V. S.; Perez L. Antibodies to Combat Viral Infections: Development Strategies and Progress. Nat. Rev. Drug Discovery 2022, 21 (9), 676–696. 10.1038/s41573-022-00495-3. 35725925 PMC9207876

[ref2] Boyoglu-Barnum S.; Ellis D.; Gillespie R. A.; Hutchinson G. B.; Park Y.-J.; Moin S. M.; Acton O. J.; Ravichandran R.; Murphy M.; Pettie D.; Matheson N.; Carter L.; Creanga A.; Watson M. J.; Kephart S.; Ataca S.; Vaile J. R.; Ueda G.; Crank M. C.; Stewart L.; Lee K. K.; Guttman M.; Baker D.; Mascola J. R.; Veesler D.; Graham B. S.; King N. P.; Kanekiyo M. Quadrivalent Influenza Nanoparticle Vaccines Induce Broad Protection. Nature 2021, 592 (7855), 623–628. 10.1038/s41586-021-03365-x. 33762730 PMC8269962

[ref3] Steichen J. M.; Lin Y.-C.; Havenar-Daughton C.; Pecetta S.; Ozorowski G.; Willis J. R.; Toy L.; Sok D.; Liguori A.; Kratochvil S.; Torres J. L.; Kalyuzhniy O.; Melzi E.; Kulp D. W.; Raemisch S.; Hu X.; Bernard S. M.; Georgeson E.; Phelps N.; Adachi Y.; Kubitz M.; Landais E.; Umotoy J.; Robinson A.; Briney B.; Wilson I. A.; Burton D. R.; Ward A. B.; Crotty S.; Batista F. D.; Schief W. R. A Generalized HIV Vaccine Design Strategy for Priming of Broadly Neutralizing Antibody Responses. Science 2019, 366 (6470), eaax4380 10.1126/science.aax4380. 31672916 PMC7092357

[ref4] Lanzavecchia A.; Frühwirth A.; Perez L.; Corti D. Antibody-Guided Vaccine Design: Identification of Protective Epitopes. Curr. Opin. Immunol. 2016, 41, 62–67. 10.1016/j.coi.2016.06.001. 27343848

[ref5] Anasir M. I.; Poh C. L. Structural Vaccinology for Viral Vaccine Design. Front. Microbiol. 2019, 10, 738 10.3389/fmicb.2019.00738. 31040832 PMC6476906

[ref6] Mba I. E.; Sharndama H. C.; Anyaegbunam Z. K. G.; Anekpo C. C.; Amadi B. C.; Morumda D.; Doowuese Y.; Ihezuo U. J.; Chukwukelu J. U.; Okeke O. P. Vaccine Development for Bacterial Pathogens: Advances, Challenges and Prospects. Trop. Med. Int. Health 2023, 28 (4), 275–299. 10.1111/tmi.13865. 36861882

[ref7] Pushparaj P.; Nicoletto A.; Sheward D. J.; Das H.; Castro Dopico X.; Perez Vidakovics L.; Hanke L.; Chernyshev M.; Narang S.; Kim S.; Fischbach J.; Ekström S.; McInerney G.; Hällberg B. M.; Murrell B.; Corcoran M.; Karlsson Hedestam G. B. Immunoglobulin Germline Gene Polymorphisms Influence the Function of SARS-CoV-2 Neutralizing Antibodies. Immunity 2023, 56 (1), 193–206.e7. 10.1016/j.immuni.2022.12.005. 36574772 PMC9742198

[ref8] Hanke L.; Sheward D. J.; Pankow A.; Vidakovics L. P.; Karl V.; Kim C.; Urgard E.; Smith N. L.; Astorga-Wells J.; Ekström S.; Coquet J. M.; McInerney G. M.; Murrell B. Multivariate Mining of an Alpaca Immune Repertoire Identifies Potent Cross-Neutralizing SARS-CoV-2 Nanobodies. Sci. Adv. 2022, 8 (12), eabm0220 10.1126/sciadv.abm0220. 35333580 PMC8956255

[ref9] Ständer S.; R Grauslund L.; Scarselli M.; Norais N.; Rand K. Epitope Mapping of Polyclonal Antibodies by Hydrogen-Deuterium Exchange Mass Spectrometry (HDX-MS). Anal. Chem. 2021, 93 (34), 11669–11678. 10.1021/acs.analchem.1c00696. 34308633

[ref10] Chowdhury S.; Gomez Toledo A.; Hjortswang E.; Sorrentino J. T.; Lewis N. E.; Bläckberg A.; Ekström S.; Izadi A.; Nordenfelt P.; Malmström L.; Rasmussen M.; Malmström J. Dissecting the Properties of Circulating IgG against Group A Streptococcus through a Combined Systems Antigenomics-Serology Workflow. bioRxiv 2023, 2023.11.07.565977 10.1101/2023.11.07.565977.

[ref11] Ozohanics O.; Ambrus A. Hydrogen-Deuterium Exchange Mass Spectrometry: A Novel Structural Biology Approach to Structure, Dynamics and Interactions of Proteins and Their Complexes. Life 2020, 10 (11), 286 10.3390/life10110286. 33203161 PMC7696067

[ref12] Yu C.; Huang L. Cross-Linking Mass Spectrometry: An Emerging Technology for Interactomics and Structural Biology. Anal. Chem. 2018, 90 (1), 144–165. 10.1021/acs.analchem.7b04431. 29160693 PMC6022837

[ref13] Mintseris J.; Gygi S. P. High-Density Chemical Cross-Linking for Modeling Protein Interactions. Proc. Natl. Acad. Sci. U. S. A. 2020, 117 (1), 93–102. 10.1073/pnas.1902931116. 31848235 PMC6955236

[ref14] Hauri S.; Khakzad H.; Happonen L.; Teleman J.; Malmström J.; Malmström L. Rapid Determination of Quaternary Protein Structures in Complex Biological Samples. Nat. Commun. 2019, 10 (1), 192 10.1038/s41467-018-07986-1. 30643114 PMC6331586

[ref15] Bahnan W.; Happonen L.; Khakzad H.; Kumra Ahnlide V.; de Neergaard T.; Wrighton S.; André O.; Bratanis E.; Tang D.; Hellmark T.; Björck L.; Shannon O.; Malmström L.; Malmström J.; Nordenfelt P. A Human Monoclonal Antibody Bivalently Binding Two Different Epitopes in Streptococcal M Protein Mediates Immune Function. EMBO Mol. Med. 2022, 15, e16208 10.15252/emmm.202216208. 36507602 PMC9906385

[ref16] Lenz S.; Sinn L. R.; O’Reilly F. J.; Fischer L.; Wegner F.; Rappsilber J. Reliable Identification of Protein-Protein Interactions by Crosslinking Mass Spectrometry. Nat. Commun. 2021, 12 (1), 3564 10.1038/s41467-021-23666-z. 34117231 PMC8196013

[ref17] Avire N. J.; Whiley H.; Ross K. A Review of Streptococcus Pyogenes: Public Health Risk Factors, Prevention and Control. Pathogens 2021, 10 (2), 248 10.3390/pathogens10020248. 33671684 PMC7926438

[ref18] Chiarot E.; Faralla C.; Chiappini N.; Tuscano G.; Falugi F.; Gambellini G.; Taddei A.; Capo S.; Cartocci E.; Veggi D.; Corrado A.; Mangiavacchi S.; Tavarini S.; Scarselli M.; Janulczyk R.; Grandi G.; Margarit I.; Bensi G. Targeted Amino Acid Substitutions Impair Streptolysin O Toxicity and Group A Streptococcus Virulence. Mbio 2013, 4 (1), e00387–12 10.1128/mbio.00387-12. PMC3546560 23300245

[ref19] van Pee K.; Neuhaus A.; D’Imprima E.; Mills D. J.; Kühlbrandt W.; Yildiz O. ¨. CryoEM Structures of Membrane Pore and Prepore Complex Reveal Cytolytic Mechanism of Pneumolysin. Elife 2017, 6, e23644 10.7554/elife.23644. 28323617 PMC5437283

[ref20] Uchiyama S.; Döhrmann S.; Timmer A. M.; Dixit N.; Ghochani M.; Bhandari T.; Timmer J. C.; Sprague K.; Bubeck-Wardenburg J.; Simon S. I.; Nizet V. Streptolysin O Rapidly Impairs Neutrophil Oxidative Burst and Antibacterial Responses to Group A Streptococcus. Front. Immunol. 2015, 6, 581 10.3389/fimmu.2015.00581. 26635795 PMC4644796

[ref21] Azuar A.; Jin W.; Mukaida S.; Hussein W. M.; Toth I.; Skwarczynski M. Recent Advances in the Development of Peptide Vaccines and Their Delivery Systems against Group A Streptococcus. Vaccines 2019, 7 (3), 58 10.3390/vaccines7030058. 31266253 PMC6789462

[ref22] Limbago B.; Penumalli V.; Weinrick B.; Scott J. R. Role of Streptolysin O in a Mouse Model of Invasive Group A Streptococcal Disease. Infect. Immun. 2000, 68 (11), 6384–6390. 10.1128/IAI.68.11.6384-6390.2000. 11035749 PMC97723

[ref23] Davies M. R.; McIntyre L.; Mutreja A.; Lacey J. A.; Lees J. A.; Towers R. J.; Duchêne S.; Smeesters P. R.; Frost H. R.; Price D. J.; Holden M. T. G.; David S.; Giffard P. M.; Worthing K. A.; Seale A. C.; Berkley J. A.; Harris S. R.; Rivera-Hernandez T.; Berking O.; Cork A. J.; Torres R. S. L. A.; Lithgow T.; Strugnell R. A.; Bergmann R.; Nitsche-Schmitz P.; Chhatwal G. S.; Bentley S. D.; Fraser J. D.; Moreland N. J.; Carapetis J. R.; Steer A. C.; Parkhill J.; Saul A.; Williamson D. A.; Currie B. J.; Tong S. Y. C.; Dougan G.; Walker M. J. Atlas of Group A Streptococcal Vaccine Candidates Compiled Using Large-Scale Comparative Genomics. Nat. Genet. 2019, 51 (6), 1035–1043. 10.1038/s41588-019-0417-8. 31133745 PMC6650292

[ref24] Walkinshaw D. R.; Wright M. E. E.; Mullin A. E.; Excler J.-L.; Kim J. H.; Steer A. C. The Streptococcus Pyogenes Vaccine Landscape. npj Vaccines 2023, 8 (1), 16 10.1038/s41541-023-00609-x. 36788225 PMC9925938

[ref25] Farrand A. J.; LaChapelle S.; Hotze E. M.; Johnson A. E.; Tweten R. K. Only Two Amino Acids Are Essential for Cytolytic Toxin Recognition of Cholesterol at the Membrane Surface. Proc. Natl. Acad. Sci. U. S. A. 2010, 107 (9), 4341–4346. 10.1073/pnas.0911581107. 20145114 PMC2840085

[ref26] Kucinskaite-Kodze I.; Simanavicius M.; Dapkunas J.; Pleckaityte M.; Zvirbliene A. Mapping of Recognition Sites of Monoclonal Antibodies Responsible for the Inhibition of Pneumolysin Functional Activity. Biomol 2020, 10 (7), 1009 10.3390/biom10071009. PMC7408604 32650398

[ref27] Feil S. C.; Ascher D. B.; Kuiper M. J.; Tweten R. K.; Parker M. W. Structural Studies of Streptococcus Pyogenes Streptolysin O Provide Insights into the Early Steps of Membrane Penetration. J. Mol. Biol. 2014, 426 (4), 785–792. 10.1016/j.jmb.2013.11.020. 24316049 PMC4323271

[ref28] Happonen L.; Hauri S.; Svensson Birkedal G.; Karlsson C.; de Neergaard T.; Khakzad H.; Nordenfelt P.; Wikström M.; Wisniewska M.; Björck L.; Malmström L.; Malmström J. A Quantitative Streptococcus Pyogenes-Human Protein-Protein Interaction Map Reveals Localization of Opsonizing Antibodies. Nat. Commun. 2019, 10 (1), 2727 10.1038/s41467-019-10583-5. 31227708 PMC6588558

[ref29] Seffernick J. T.; Lindert S. Hybrid Methods for Combined Experimental and Computational Determination of Protein Structure. J. Chem. Phys. 2020, 153 (24), 240901 10.1063/5.0026025. 33380110 PMC7773420

[ref30] Ambrosetti F.; Jandova Z.; Bonvin A. M. J. J. Information-Driven Antibody–Antigen Modelling with HADDOCK. Methods Mol. Biol. 2023, 2552, 267–282. 10.1007/978-1-0716-2609-2_14. 36346597

[ref31] Ambrosetti F.; Jiménez-García B.; Roel-Touris J.; Bonvin A. M. J. J. Modeling Antibody-Antigen Complexes by Information-Driven Docking. Structure 2020, 28 (1), 119–129.e2. 10.1016/j.str.2019.10.011. 31727476

[ref32] de Vries S. J.; van Dijk M.; Bonvin A. M. J. J. The HADDOCK Web Server for Data-Driven Biomolecular Docking. Nat. Protoc. 2010, 5 (5), 883–897. 10.1038/nprot.2010.32. 20431534

[ref33] Zhang M. M.; Beno B. R.; Huang R. Y.-C.; Adhikari J.; Deyanova E. G.; Li J.; Chen G.; Gross M. L. An Integrated Approach for Determining a Protein-Protein Binding Interface in Solution and an Evaluation of Hydrogen-Deuterium Exchange Kinetics for Adjudicating Candidate Docking Models. Anal. Chem. 2019, 91 (24), 15709–15717. 10.1021/acs.analchem.9b03879. 31710208 PMC7497726

[ref34] Manthei K. A.; Patra D.; Wilson C. J.; Fawaz M. V.; Piersimoni L.; Shenkar J. C.; Yuan W.; Andrews P. C.; Engen J. R.; Schwendeman A.; Ohi M. D.; Tesmer J. J. G. Structural Analysis of Lecithin:Cholesterol Acyltransferase Bound to High Density Lipoprotein Particles. Commun. Biol. 2020, 3 (1), 28 10.1038/s42003-019-0749-z. 31942029 PMC6962161

