# Supplementary material for: Multimodal Mass Spectrometry Identifies a Conserved Protective Epitope in S. pyogenes Streptolysin O
Source: Anal Chem. 2024 May 3;96(22):9060–8. doi: 10.1021/acs.analchem.4c00596 (PMC11154737; doi:10.1021/acs.analchem.4c00596)
Supplement: Supplementary file 1 — ac4c00596_si_001.pdf [file ac4c00596_si_001.pdf]

Supporting Information

Multi-Modal Mass Spectrometry Identifies a Conserved Protective Epitope in *S. pyogenes* Streptolysin O

Di Tang[1], Carlos Gueto-Tettay[1], Elisabeth Hjortswang[1], Joel Ströbaek[1], Simon Ekström[2], Lotta Happonen[1], Lars Malmström[1] & Johan Malmström[1*]

1. Division of Infection Medicine, Department of Clinical Sciences, Faculty of Medicine, Lund University, Klinikgatan 32, 222 42 Lund, Sweden

2. SciLifeLab, Integrated Structural Biology platform, Structural Proteomics Unit Sweden, Lund University, Klinikgatan 32, 222 42 Lund, Sweden

*Correspondence should be addressed to: Dr. Johan Malmström (johan.malmstrom@med.lu.se)
BMC, D13
Department of Clinical Sciences, Faculty of Medicine
Lund University
Sölvegatan 19, SE-221 84 Lund, Sweden
Telephone: Int. +46 46-222 08 30

Table of Contents

- Supplementary Figure 1-2
- Supplementary Table 1-6
- Supplementary Methods
- Data availability
- Supplementary References

Supplementary Figures

Supplementary Figure 1: Tang et al

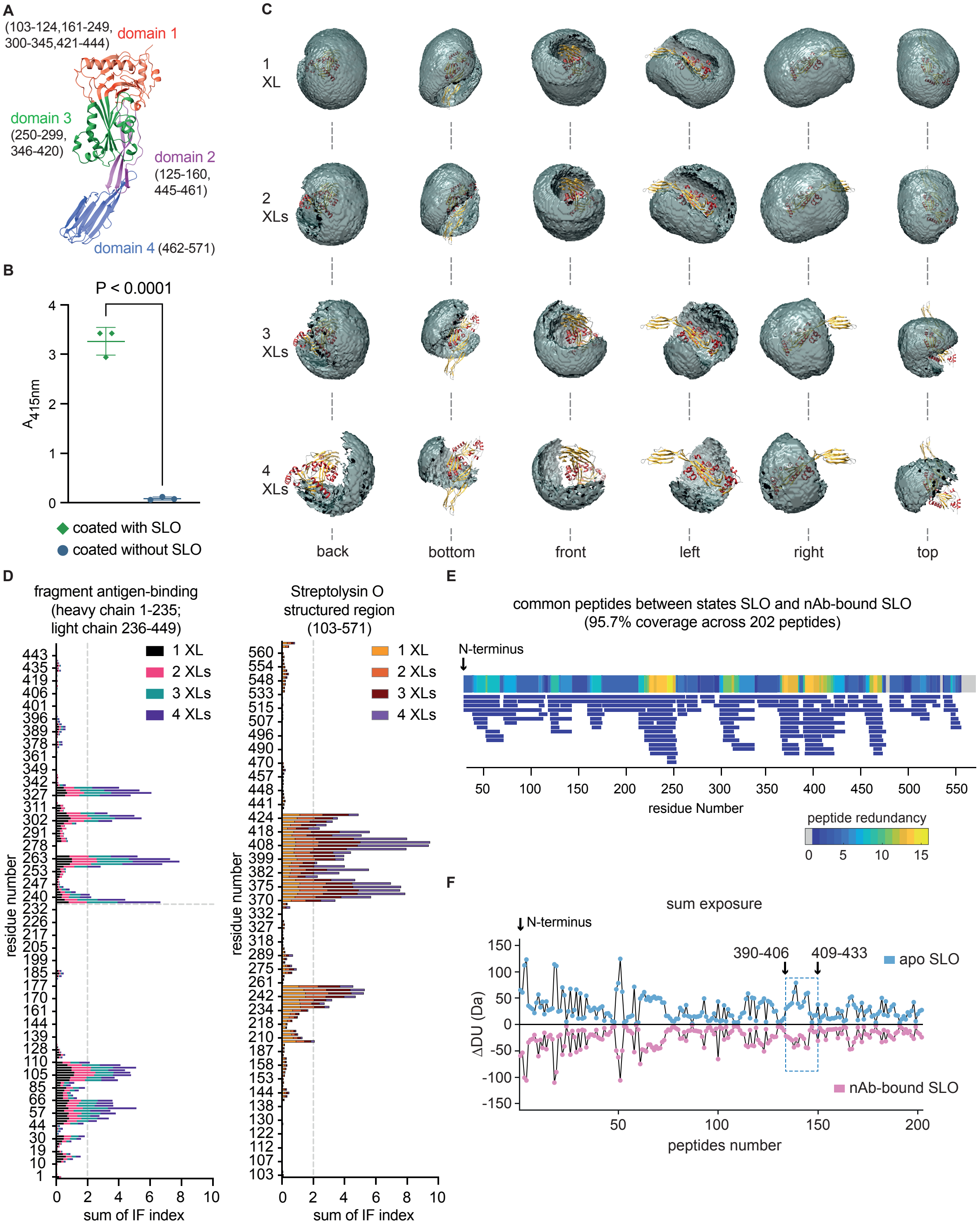

Figure S1A). Annotated SLO domains with residue numbers, colored respectively. **B).** The ELISA result for monoclonal antibody binding specificity against SLO, unpaired t-test analysis was conducted to compare conditions. **C).** Six views of the Fab interaction space directing the SLO protein presented in cartoon, constructed by any one to all four distance constraints via the DisVis [1] complete analysis mode.

D). DisVis interaction analysis showing residue IF at the interface within all possible Fab-SLO pairwise complex conformation, consistent with at least 1 to 4 distance constraints. **E).** The redundancy map of 202 common peptides identified in both apo SLO and nAb-SLO complex from the HDX-MS experiments. Peptide fragments are displayed as bar with the length correlating to peptide length, with residue redundancy visualized by a color gradient from 0 to 16. **F).** The cumulative Da changes due to deuterium uptake across all shared peptides are shown in a butterfly plot. Peptide region with differential deuterium uptake are framed by dashed lines. First and last peptides from that protected region are labeled with residue numbers accordingly.

Supplementary Figure 2: Tang et al

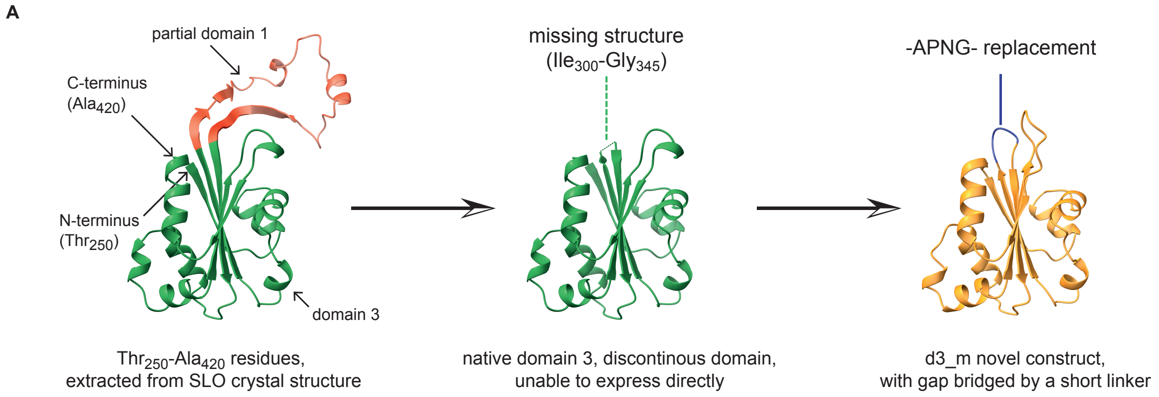

Figure S2A). The redesign process of the novel d3_m construct (orange) to mimic the protective epitope located in the native domain 3 (green) of SLO protein.

Supplementary Tables

XL no.	Peptide1	Peptide2	Protein1	Protein2	CSM count
1	DIQMTQTTSSLSASLGDR(1)	IAPGDGDPFYNKMFTYK(12)	Fab LC	Fab HC	188
2	DSTYSMSSTLTLTKDEYER(14)	TSTSPIVKSFNR(8)	Fab LC	Fab LC	14
3	YLNWYQQKPDGTLK(8)	ASQDIKK(6)	Fab LC	Fab LC	12
4	TSTSPIVKSFNR(8)	WKIDGSER(2)	Fab LC	Fab LC	9
5	DIQMTQTTSSLSASLGDR(1)	ASQDIKK(6)	Fab LC	Fab LC	3
6	HNSYTCEATHKTSTSPIVK(11)	WKIDGSER(2)	Fab LC	Fab LC	2
7	QNGVLNSWTDQDSKDSTYSMSSTLTLTK(14)	YLNWYQQKPDGTLK(8)	Fab LC	Fab LC	1

Table S1. The summary of DSS cross-linked peptide pairs found in nAb Fab domain by pLink2 [2]. Cross-linked residue positions are indicated in round brackets. LC: light chain; HC: heavy chain; CSM: cross-linked peptide spectrum match.

XL no.	linker	Peptide1	Peptide2	Protein1	Protein2	CSM count	MS/MS spectra
**1**	**DSG**	**YSDILENSSFTAVVLGGDAAEHNKVVTK(24)**	**IAPGDGDPFYNKMFTYK(12)**	**SLO**	**Fab HC**	**20**	**Fig.2E**
2	DSG	KYLNWYQQKPDGTLK(1)	GTDVKTNGK(5)	Fab LC	SLO	13	
3	DSG	TSTSPIVKSFNR(8)	KVIDER(1)	Fab LC	SLO	1	
**4**	**DSS**	**KYLNWYQQKPDGTLK(1)**	**GTDVKTNGK(5)**	**Fab LC**	**SLO**	**15**	**Fig.2E**
5	DSS	IAPGDGDPFYNKMFTYK(12)	KNPAYPISYTSVFLK(1)	Fab HC	SLO	4	
6	DSS	IAPGDGDPFYNKMFTYK(12)	GFTENKPDAVVTK(6)	Fab HC	SLO	3	
7	DSS	KYLNWYQQKPDGTLK(1)	DVKLSK(3)	Fab LC	SLO	3	
8	DSS	KYLNWYQQKPDGTLK(1)	KVIDER(1)	Fab LC	SLO	2	
9	DSS	TSTSPIVKSFNR(8)	KVIDER(1)	Fab LC	SLO	2	
10	DSS	YSDILENSSFTAVVLGGDAAEHNKVVTK(24)	IAPGDGDPFYNKMFTYK(12)	SLO	Fab HC	2	
11	DSS	NVIKDNATFSR(4)	ASQDIKK(6)	SLO	Fab LC	1	
12	DSS	DIQMTOTTSSLSASLGDR(1)	NVIKDNATFSR(4)	Fab LC	SLO	1	

Table S2. The summary of DSG or DSS cross-linked peptide pairs found between nAb Fab domain and SLO. The two most representative cross-links are highlighted in bold. No.12 cross-link was removed for unspecific interaction.

103 104 105 106 107 108 110 111 112 115 117 119 122 126 127 128 130 132 133 137 138 139 141 142 144 145 150 152 153 154 155 157 158 159 160 183 187 188 201 208 210 211 212 216 218 224 227 231 234 238 239 241 242 243 244 245 261 268 270 272 275 283 286 287 289 307 310 311 318 319 323 326 327 328 329 331 332 333 357 359 370 371 372 373 375 376 377 379 382 385 386 397 399 400 402 407 408 410 414 417 418 419 421 423 424 425 439 440 441 442 445 447 448 451 454 456 457 458 459 464 470 485 488 489 490 491 492 495 496 501 502 506 507 509 511 513 515 518 519 521 533 544 545 546 548 550 551 552 554 556 558 559 560 561 569 571
1 3 5 8 9 10 13 14 15 17 19 23 25 26 28 30 31 41 42 43 44 53 54 55 56 57 61 62 63 65 66 71 74 75 84 85 88 89 103 104 105 106 107 108 109 110 118 125 126 127 128 129 130 131 138 139 140 141 142 143 144 145 146 147 148 161 162 164 168 169 170 171 173 174 175 177 178 180 182 184 185 186 195 197 198 199 200 202 203 204 205 211 213 214 216 217 218 220 222 225 226 227 228 229 231 232 234 235 236 238 240 242 243 244 245 247 249 250 251 252 253 255 259 261 262 263 265 275 276 277 278 279 287 288 289 291 292 295 298 300 302 303 304 305 309 311 312 314 315 316 327 328 329 330 335 342 343 344 345 347 349 354 356 357 358 361 362 363 373 377 378 380 386 387 388 389 391 392 393 395 396 397 398 399 400 401 402 403 404 405 406 413 415 417 418 419 422 423 425 434 435 437 438 439 441 443 445 447 448 449

Table S3. Accessible residue input for DisVis interaction/complete analysis, which should be prepared in .txt format. Putative interactive residue candidates were filtered by a relative solvent accessibility (RSA) value > 40%, predicted by NetSurfP-3.0 [3] for both SLO (top panel) and Fab (bottom panel) sequences.

Fixed chain	Res. no.	Atom	Scanning chain	Res. no.	Atom	Min. distance (Å)	Max. distance (Å)
A	375	CA	B	266	CA	9	24
A	403	CA	A	62	CA	9	24
A	417	CA	B	265	CA	10	30
A	425	CA	A	62	CA	10	30

Table S4. Distance constraint input for DisVis interaction analysis, which should be prepared in .txt format. "Fixed chain" is set to be single chain SLO molecule 1, as "A"; "Scanning chain" is set to be the modeled Fab domain molecule 2, with "B" representing light chain or "A" representing heavy chain. Res.no.: residue number. Fab residues are consecutively renumbered as heavy chain 1-235 and light chain 236-449. CA: alpha carbon atom. Distance range as "Min. distance" and "Max. distance" was set according to previous studies [4,5].

Cluster 1	
HADDOCK score	-139.3 +/- 4.0
Cluster size	240
RMSD from the overall lowest-energy structure	1.7 +/- 1.7
Van der Waals energy	-79.0 +/- 2.8
Electrostatic energy	-371.1 +/- 26.7
Desolvation energy	3.9 +/- 1.1
Restraints violation energy	100.6 +/- 41.94
Buried Surface Area	2335.3 +/- 91.6
Z-Score	-1.4
**Cluster 3**	
HADDOCK score	-130.6 +/- 5.4
Cluster size	62
RMSD from the overall lowest-energy structure	5.1 +/- 0.2
Van der Waals energy	-61.1 +/- 5.5
Electrostatic energy	-409.1 +/- 45.3
Desolvation energy	0.4 +/- 1.5
Restraints violation energy	118.8 +/- 23.74
Buried Surface Area	2524.5 +/- 90.9
Z-Score	-1.0
**Cluster 5**	
HADDOCK score	-88.6 +/- 12.8
Cluster size	4
RMSD from the overall lowest-energy structure	18.5 +/- 0.2
Van der Waals energy	-53.5 +/- 8.6
Electrostatic energy	-273.0 +/- 17.7
Desolvation energy	10.9 +/- 1.9
Restraints violation energy	84.9 +/- 57.67
Buried Surface Area	2042.7 +/- 184.2
Z-Score	0.6

Table S5. The summary of modeled Fab-SLO pairwise complexes using HADDOCK 2.4 antibody-antigen docking protocol [6–8]. 394 generated Fab-SLO structures were clustered into 5 groups, representing 98.5% of the final water-refined models. Top three clusters are shown here as above, the top-ranked Fab-SLO model (determined by the lowest HADDOCK combined score as -145) from the cluster 1, also as the largest cluster, was used for following analysis (including both PRODIGY [9] and SpotOn [10]) to identify the interface residues.

hotspots												
Res.no.	245	346	372	393	402	410	414	415	417	418	419	421
AA	THR	ARG	THR	VAL	ASN	ASP	ASN	VAL	LYS	ASP	ASN	THR

nullspots													
Res.no.	104	243	244	246	247	248	373	375	391	392	395	399	400
AA	ASP	GLY	ASN	LEU	PRO	ALA	ASP	LYS	ALA	VAL	GLY	ALA	GLU
401	403	404	405	408	411	413	420	422	423	424	425	426	429
HIS	LYS	VAL	VAL	ASP	VAL	ARG	ALA	PHE	SER	ARG	LYS	ASN	TYR

Table S6. List of hotspots (most important interface residues) and nullspots (non-hotspot residues) on SLO protein, predicted by SpotOn [10] within the top-ranked Fab-SLO pairwise complex model. AA: amino acid.

Supplementary Methods

Protein production and purification

The complete SLO sequence (Uniprot ID: P0DF96), excluding the signal peptide, was engineered to incorporate an N-terminal Strep-HA-His tag. The plasmid carrying the tag-SLO gene was synthesized and assembled by the Lund University Protein Production Platform, subsequently transformed into BL21(DE3) Competent Cells (Thermo Scientific). The induction and purification of the target tag-SLO protein was carried out in-house following a previously published protocol [11]. The reverse-engineered d3_m construct was expressed and purified by the Protein Production Sweden Umeå node.

Monoclonal antibody binding specificity and its neutralization against SLO hemolysis

For ELISA assays, an equivalent quantity of SLO protein or the d3_m construct (ca. 3 μg) was immobilized onto a MaxiSorp plate (Thermo Scientific), which was subsequently incubated with either 0.4 μg nAb (followed by serial half-dilution), Xolair or 1x PBS (Phosphate buffer saline tablet, Sigma Aldrich) as a background control. Post thorough washing with PBST buffer (1x PBS, 0.1% Tween 20), a secondary HRP-conjugated anti-mouse IgG goat antibody (Bio-Rad) and HRP substrate kit (Bio-Rad) were applied in sequence. After 3 min development, the plate was read in a microplate reader (BMG Labtech) at a wavelength of 415 nm. To compare binding of the nAb against full length SLO and d3_m construct, Prism 10 built-in non-linear regression (Equation: One site -- Specific binding) mode was applied to derive the K_d and B_{max} with 95% confidence interval. For the SLO cytolysis inhibition assay, sheep red blood cells (Thermo Scientific, Oxoid) were first diluted with 1x PBS accordingly [12], followed by the addition of 0.1 μg of active SLO protein reduced by TCEP (Sigma Aldrich) and 5 μg of corresponding IgG or different IgG fragments. Xolair was purchased from Novartis. P.IgG represents a pool of immunoglobulin G isolated from the plasma of a donor who recently recovered from a GAS infection. A FragIT kit (Genovis) was utilized to digest P.IgG and generate two fractions: F(ab')2- and Fc-fragments. Following incubation in ThermoMixer (Eppendorf) at 37°C and 300 rpm for 30 minutes, the plates were centrifuged, and the supernatant was transferred to a new plate for reading at 541 nm wavelength by a microplate reader (BMG Labtech). Measured absorbance value correlates to the quantity of leaked hemoglobin due to hemolysis. The positive control using SLO alone was defined as a 100% lysis rate.

De novo sequencing of the nAb, assembling protein and modeling of the antibody Fab

The full-length nAb was initially reduced with 5 mM TCEP (Sigma Aldrich) and alkylated with 10 mM IAA (Sigma Aldrich), followed by overnight digestion in ThermoMixer (Eppendorf) at 37°C and 500 rpm, using trypsin, chymotrypsin, elastase, and pepsin (Promega) at an enzyme to substrate ratio of 1:20. The digested peptides were then purified using a C18 clean-up spin column (Thermo Scientific), concentrated in a SpeedVac (Eppendorf), and reconstituted into buffer A (2% acetonitrile, 0.2% formic acid) prior to mass spectrometry analysis.

Approximately 1 μg of peptides from each sample, quantified using a NanoDrop spectrophotometer (DeNovix), were loaded onto an EASY-nLC 1200 system interfaced with a Q Exactive HF-X hybrid quadrupole-Orbitrap mass spectrometer (Thermo Scientific). Each enzyme-digested sample was analyzed in duplicate injection. The peptides were first concentrated on a precolumn (PepMap100 C18 3 μm; 75 μm × 2 cm; Thermo Fisher Scientific) and then separated on an EASY-Spray column (ES903, column temperature 45 °C; Thermo Fisher Scientific), in according with the manufacturer recommendations. Two solvents were used as mobile phases: solvent A (0.1% formic acid) and solvent B (0.1% formic acid, 80% acetonitrile). A linear gradient from 5 to 38% B was employed over 180 minutes at a constant flow rate of 350 nl/min. For data acquisition, a data-dependent acquisition (DDA) method was implemented as follows. An initial MS1 scan with a scan range of 350–1650 m/z, resolution of 120,000, auto gain control (AGC) target of $3e^6$ and maximum IT (injection time) 45 ms was followed by the top 15 MS2 scans at a resolution of 15,000, AGC target $1e^5$, 30 ms IT and a stepped normalized collision energy (NCE) of 20, 25 and 30. Charge states of 1, 6-8 and above were excluded, except in samples digested with other enzymes than trypsin where singly charged ions were included. The performance of the LC-MS system was controlled by

analyzing a yeast protein extract digest (Promega). A total of eight datasets were collected and subsequently processed using multiple *de novo* MS sequencing algorithms [13].

A novel approach involving cumulative fragment-ion evidence was applied to enhance *de novo* peptide sequencing and subsequent primary protein structure assembly. Peptide candidates were generated using three deep-learning-based *de novo* peptide tools: PointNovo [14], CasaNovo [15], and InstaNovo [16]. For PointNovo, two in-house multienzyme-trained models [17] were utilized, whereas default models were employed for CasaNovo and InstaNovo. The study considered twelve fragment ions: a^{+1}, a^{+2}, b^{+1}, b^{+2}, y^{+1}, y^{+2}, a-H2O, b-H2O, y-H2O, a-NH3, b-NH3, and y-NH3, with candidate selection based on a 20 ppm tolerance at both MS1 and MS2 levels and the observation of a minimum of four fragment ions. A positional evidence vector with length L was constructed for each candidate, filled by the count of found fragment ions at each position. These vectors were then pooled across all spectra from all samples.

Subsequently, peptides were segmented into 5-mers retaining the pooled positional fragment ion information. Cumulative MS evidence for these new 5-mer vectors was compiled elementwise. Finally, a cumulative MS score (cMS) for each peptide candidate was calculated by condensing the positional ion global evidence and normalizing for peptide length. This MS-evidence-driven strategy effectively disseminates information on redundant and overlapping segments identified across all MS samples analyzed. The highest-ranked peptide candidates were those that not only showed maximal overlap but also robust MS evidence supporting the target protein sequences. The positional confidence score [17] for the assembled heterodimeric chains of the Fab domain is illustrated in **Figure 1E**.

Next, proABC-2 was employed to predict the hypervariable region of the nAb Fab domain, of relevance for subsequent docking studies [18]. This was informed by the previously acquired sequences of both the heavy and light chains of the nAb. The structure of the nAb Fab fragment was predicted with AlphaFold-Multimer (v2.3.1) [19] using the version-specific Docker container with default settings. The resulting structures were verified by calculating the inter-chain pDockQ values for each model [20]. The predicted model with the highest pDockQ value was used for downstream analysis.

Cross-linking mass spectrometry and data analysis

The exploratory modeling process involved XL-MS for an in-solution cross-linking reaction and subsequent cross-linked peptide identification. The nAb and SLO were mixed at a 1:1 molar ratio in 1x PBS solution at 37°C with 500 rpm agitation for 1 hour incubation. The duplet linkers, DSS-H12/D12 or DSG-H6/D6 (Creative Molecules), were then added to cross-link the samples over the course of two hours. The reaction was quenched with 4 M ammonia bicarbonate (Sigma Aldrich), and followed by a standard reduction and alkylation procedures as stated above. We then employed a two-step digestion process (involving lysyl endopeptidase, from FUJIFILM Wako Chemicals U.S.A. Corporation, followed by trypsin, Promega) in-solution to generate the cross-linked peptide pairs.

The peptides were cleaned, dried, and reconstituted before analysis by mass spectrometer as using the same protocol described above. Approximately 800 ng of the peptides from each sample, quantified using a NanoDrop spectrophotometer, were loaded into an Ultimate 3000 UPLC system connected to an Orbitrap Eclipse Tribrid Mass Spectrometer (Thermo Scientific). Each sample was performed technical duplicate injections. Column equilibration and sample loading were performed according to the manufacturer guidelines. The mobile phases used included Solvent A (0.1% formic acid) and Solvent B (0.1% formic acid and 80% acetonitrile). The gradient was linear and ranged from 5 to 38%, with a consistent flow rate of 300 nl/min over 90 minutes. The DDA method consisted of one MS1 scan with a scan range of 350–1650 m/z, resolution of 120,000, a standard-mode AGC target and auto-mode maximum injection time. The fragment setup included a 3-second cycle time with MS2 scans, 15,000 resolution, standard AGC target, 22 ms IT and NCE of 30. All runs incorporated charge states from 2 to 6. The LC-MS performance was monitored prior to analysis using HeLa protein digest standard (Thermo Fisher Scientific).

The cross-linking datasets were analyzed using pLink2 [2], configuring DSG-H6/D6 and DSS-H12/D12 (Creative Molecules Inc.) linker modifications according to the manufacturer product pages. The searched library included the sequences for the heavy, light chains of nAb and SLO along with common

contaminants, and a maximum of two missed cleavage sites were allowed. Visualization of the top two nAb-SLO crosslinked peptides was done by xiSPEC [21], based on the corresponding extracted peak lists.

DisVis interaction analysis based on distance constraints generated by XL-MS

All crosslinked peptides that connected the nAb Fab fragment and SLO, identified from both cross-linking experiments with duplet linkers, were summarized. DisVis was employed for quick scanning and interaction analysis [1], utilizing the crystal structure of SLO and the modeled Fab structure for exploratory modeling. To begin with, the distance between Cα-Cα derived from the inter-protein cross-linked residues was set within a range of 0-30 Å. Initially, all identified XL constraints were used to calculate the z-score and group the clusters. The cluster comprising four XLs, which included the two most abundant XLs, was further utilized in the interaction analysis, with more stringent range of Cα-Cα distance as elaborated in supplementary table 4. The prediction of accessible residues of both Fab and SLO was conducted using NetSurfP-3.0 [3], focusing on those with a relative solvent accessibility (RSA) value greater than 40%. The interaction fraction index was computed through the interaction analysis mode of DisVis [1], designating consistent IF values greater than 0.5 as putative residues that contribute to forming the suggested Fab-SLO binding interface. Above cut-off values were set according to DisVis developer manual to maximize confidence and reliability of the modeling practice. A step-by-step tutorial recommended by the developers could be found here, https://www.bonvinlab.org/education/Others/disvis-webserver/.

Distance-information-driven docking of Fab-SLO protein complex by HADDOCK

The HADDOCK 2.4 antibody-antigen information-driven docking protocol was implemented to construct the most accurate possible complex and to identify relevant interface contact residues [6–8]. Both the modeled Fab structure and the crystal structure of SLO were applied, with the predicted HV loops of nAb Fab fragment assigned as active residues, and DisVis-distance-derived putative interactive residues of SLO designated as passive residues. Two prevalent crosslink sites with a Cα-Cα range of 0-30Å were established as the center of mass constraints to enforce contact. Separate sampling parameters were set for rigid body docking, semi-flexible refinement, and final refinement at 10000, 400, and 400 models, respectively. Step-by-step tutorials recommended by the developers could be found as below,
https://www.bonvinlab.org/education/HADDOCK24/HADDOCK24-CACA-guided/,
https://www.bonvinlab.org/education/HADDOCK24/HADDOCK24-antibody-antigen/.
From the final refined candidate models, the one with the lowest combined HADDOCK score (indicating highest confidence) was selected for downstream interface extraction following manual inspection. Finally, PRODIGY [9] was employed to classify the corresponding types of interaction within the interface, and SpotON [10] to predict "hotspot" residues, which are presumed to be considerably involved in intermolecular interactions.

HDX-MS experiment and data analysis

The HDX-MS experimental setup involved a LEAP H/D-X PAL™ platform for automated sample preparation, which was interfaced with an LC-MS system that consisted of an Ultimate 3000 micro-LC connected to an Orbitrap Q Exactive Plus MS. HDX was carried out on SLO protein, both with and without commercially obtained nAb, in 10 mM PBS. Apo state (unbound SLO) and epitope mapping (Ab-bound SLO) samples were incubated for t = 0, 60, 1800, 9000 seconds at 20 °C in either PBS or an HDX labelling buffer of identical composition prepared in D_2O. The experiment was conducted in a single, continuous run, with three replicates undertaken for each state and timepoint. The labelling reaction was quenched through dilution with 1% TFA, 0.4 M TCEP, 4 M urea, at 4 °C. The quenched sample was directly injected and subjected to online pepsin digestion at 4 °C. A flow of 50 µL/min 0.1% formic acid was applied for 4 minutes for online digestion and trapping of the samples. Digestion products underwent online solid phase extraction and washing with 0.1% FA for 60s on a trap column (PepMap300 C18), which was switched in-line with a reversed-phase analytical column (Hypersil GOLD). Separation occurred at 1 °C, with mobile phases of 0.1% formic acid (A) and 95% acetonitrile/0.1% formic acid (B), using a gradient of 5-50% B over 8 minutes and then from 50 to 90% B for 5 minutes. The separated peptides were analyzed on a Q

Exactive Plus MS, equipped with a heated electrospray source (HESI) operating at a capillary temperature of 250 °C with sheath gas 12 au, auxiliary gas 2 au, and sweep gas 1 au. For HDX analysis, MS full scan spectra were obtained at 70,000 resolution, automatic gain control $3e^6$, Max ion injection time 200 ms and scan range 300-2000 m/z. The identification of generated peptides was performed by analyzing separate un-deuterated samples using data dependent acquisition MS/MS. A library pool of peptides that included peptide sequence, charge state, and retention time was curated for the HDX analysis by running pepsin-digested, un-deuterated samples against the SLO sequence on PEAKS Studio X (Bioinformatics Solutions Inc.). HDX data analysis and visualization were performed using HDExaminer v3.1.1 (Sierra Analytics Inc.).
Ab-bound states were analyzed compared to Apo states, using a single charge state per peptide. Given the comparative nature of the measurements, the deuterium incorporation for the peptic peptides was derived from the observed relative mass difference between the deuterated and non-deuterated peptides without back-exchange correction using a fully deuterated sample. The spectra for all time points were manually inspected; low scoring peptides, obvious outliers, and peptides for which retention time correction could not be made consistent were removed. Deuteros 2.0 [22,23] was further applied to perform the hybrid significance test and to visualize the change of deuterium uptake in coverage plot, butterfly plot, volcano plot, kinetic uptake, as well as projecting the protected peptide residue coordinates to the indicated 3D structure.

Carriage and epitope conservation analysis, and reverse-engineering of the construct
A dedicated BLAST database was established using genomic data from *Streptococcus pyogenes* sourced from The Bacterial and Viral Bioinformatics Resource Center (BV-BRC) [24] as of March 28, 2023. Genomes classified as being of poor quality, sourced from plasmids, or as duplicate entries were excluded. This led to the creation of a curated database comprising 2216 genomes, narrowed down from the original 2283 entries. Employing BLASTp [25], the Streptolysin O sequence (Uniprot ID: P0DF96) was queried against this database. Hits that covered over 98% of the query sequence were considered matches, with the threshold selected based on data characteristics. Domain 3 of Streptolysin O from *Streptococcus pyogenes* M3, spanning residues 250-299 and 346-420, was determined as the target for construct design. This domain, interrupted by a subsequence of domain 1, required a re-engineered approach for continuity. The Message Passing Neural Network ProteinMPNN [26] algorithm was utilized to determine an alternate amino acid sequence capable of maintaining the original backbone conformation of domain 3. To start with, the redesign process commenced with the substitution of the domain 1 sequence with a predefined tetra-glycine linker (-GGGG-), chosen for its structural flexibility. Then, -APNG- linker sequence was predicted for replacing domain 1 subsequence. This modified sequence's quaternary structure was then predicted using AlphaFold2 [27] and subsequently aligned to the native structure in ChimeraX to confirm that domain 3's integrity was preserved post-substitution. Additionally, ensuring the structural stability of the designed construct was paramount. An assessment was conducted to ascertain if substituting any internal amino acids could yield a more stable conformation. ProteinMPNN [26] was employed to evaluate potential replacements for the internal residues, while all surface residues remained unchanged. This optimization process revealed that the native internal residues were optimal, and no substitutions were necessary.

The workflow illustration was created by BioRender.com with an academic license. GraphPad Prism 10 was used to generate dot plots, bar lots, and heatmaps, and perform statistics and regression analysis. ChimeraX and Chimera [28] were applied to visualize corresponding cross-links as pseudo-bonds, to depict 3D structures of proteins, domains, and epitopes, and to perform superimposition and alignment.

Data availability

All mass spectrometry proteomics data (including *de novo* sequencing BU-MS, XL-MS and HDX-MS) have been deposited to the ProteomeXchange Consortium via the PRIDE [29] partner repository with the dataset identifier PXD047461.

Supplementary References

(1) Zundert, G. C. P. van; Trellet, M.; Schaarschmidt, J.; Kurkcuoglu, Z.; David, M.; Verlato, M.; Rosato, A.; Bonvin, A. M. J. J. The DisVis and PowerFit Web Servers: Explorative and Integrative Modeling of Biomolecular Complexes. *J Mol Biol* **2017**, *429* (3), 399–407. https://doi.org/10.1016/j.jmb.2016.11.032.

(2) Chen, Z.-L.; Meng, J.-M.; Cao, Y.; Yin, J.-L.; Fang, R.-Q.; Fan, S.-B.; Liu, C.; Zeng, W.-F.; Ding, Y.-H.; Tan, D.; Wu, L.; Zhou, W.-J.; Chi, H.; Sun, R.-X.; Dong, M.-Q.; He, S.-M. A High-Speed Search Engine PLink 2 with Systematic Evaluation for Proteome-Scale Identification of Cross-Linked Peptides. *Nat Commun* **2019**, *10* (1), 3404. https://doi.org/10.1038/s41467-019-11337-z.

(3) Høie, M. H.; Kiehl, E. N.; Petersen, B.; Nielsen, M.; Winther, O.; Nielsen, H.; Hallgren, J.; Marcatili, P. NetSurfP-3.0: Accurate and Fast Prediction of Protein Structural Features by Protein Language Models and Deep Learning. *Nucleic Acids Res* **2022**, *50* (W1), gkac439-. https://doi.org/10.1093/nar/gkac439.

(4) Bonvin, A. M. J. J.; Karaca, E.; Kastritis, P. L.; Rodrigues, J. P. G. L. M. Defining Distance Restraints in HADDOCK. *Nat Protoc* **2018**, *13* (7), 1503–1503. https://doi.org/10.1038/s41596-018-0017-6.

(5) Merkley, E. D.; Rysavy, S.; Kahraman, A.; Hafen, R. P.; Daggett, V.; Adkins, J. N. Distance Restraints from Crosslinking Mass Spectrometry: Mining a Molecular Dynamics Simulation Database to Evaluate Lysine–Lysine Distances. *Protein Sci* **2014**, *23* (6), 747–759. https://doi.org/10.1002/pro.2458.

(6) Ambrosetti, F.; Jandova, Z.; Bonvin, A. M. J. J. Computer-Aided Antibody Design. *Methods Mol Biology* **2022**, *2552*, 267–282. https://doi.org/10.1007/978-1-0716-2609-2_14.

(7) Ambrosetti, F.; Jiménez-García, B.; Roel-Touris, J.; Bonvin, A. M. J. J. Modeling Antibody-Antigen Complexes by Information-Driven Docking. *Structure* **2020**, *28* (1), 119-129.e2. https://doi.org/10.1016/j.str.2019.10.011.

(8) Vries, S. J. de; Dijk, M. van; Bonvin, A. M. J. J. The HADDOCK Web Server for Data-Driven Biomolecular Docking. *Nat Protoc* **2010**, *5* (5), 883–897. https://doi.org/10.1038/nprot.2010.32.

(9) Xue, L. C.; Rodrigues, J. P.; Kastritis, P. L.; Bonvin, A. M.; Vangone, A. PRODIGY: A Web Server for Predicting the Binding Affinity of Protein–Protein Complexes. *Bioinformatics* **2016**, *32* (23), 3676–3678. https://doi.org/10.1093/bioinformatics/btw514.

(10) Moreira, I. S.; Koukos, P. I.; Melo, R.; Almeida, J. G.; Preto, A. J.; Schaarschmidt, J.; Trellet, M.; Gümüş, Z. H.; Costa, J.; Bonvin, A. M. J. J. SpotOn: High Accuracy Identification of

Protein-Protein Interface Hot-Spots. *Sci. Reports* **2017**, *7* (1), 8007. https://doi.org/10.1038/s41598-017-08321-2.

(11) Feil, S. C.; Ascher, D. B.; Kuiper, M. J.; Tweten, R. K.; Parker, M. W. Structural Studies of Streptococcus Pyogenes Streptolysin O Provide Insights into the Early Steps of Membrane Penetration. *J Mol Biol* **2014**, *426* (4), 785–792. https://doi.org/10.1016/j.jmb.2013.11.020.

(12) Sierig, G.; Cywes, C.; Wessels, M. R.; Ashbaugh, C. D. Cytotoxic Effects of Streptolysin O and Streptolysin S Enhance the Virulence of Poorly Encapsulated Group A Streptococci. *Infect Immun* **2003**, *71* (1), 446–455. https://doi.org/10.1128/iai.71.1.446-455.2003.

(13) Beslic, D.; Tscheuschner, G.; Renard, B. Y.; Weller, M. G.; Muth, T. Comprehensive Evaluation of Peptide de Novo Sequencing Tools for Monoclonal Antibody Assembly. *Brief Bioinform* **2022**, *24* (1), bbac542. https://doi.org/10.1093/bib/bbac542.

(14) Qiao, R.; Tran, N. H.; Xin, L.; Chen, X.; Li, M.; Shan, B.; Ghodsi, A. Computationally Instrument-Resolution-Independent de Novo Peptide Sequencing for High-Resolution Devices. *Nat. Mach. Intell.* **2021**, *3* (5), 420–425. https://doi.org/10.1038/s42256-021-00304-3.

(15) Yilmaz, M.; Fondrie, W. E.; Bittremieux, W.; Nelson, R.; Ananth, V.; Oh, S.; Noble, W. S. Sequence-to-Sequence Translation from Mass Spectra to Peptides with a Transformer Model. *bioRxiv* **2023**, 2023.01.03.522621. https://doi.org/10.1101/2023.01.03.522621.

(16) Eloff, K.; Kalogeropoulos, K.; Morell, O.; Mabona, A.; Jespersen, J. B.; Williams, W.; Beljouw, S. P. B. van; Skwark, M.; Laustsen, A. H.; Brouns, S. J. J.; Ljungars, A.; Schoof, E. M.; Goey, J. V.; Keller, U. auf dem; Beguir, K.; Carranza, N. L.; Jenkins, T. P. De Novo Peptide Sequencing with InstaNovo: Accurate, Database-Free Peptide Identification for Large Scale Proteomics Experiments. *bioRxiv* **2023**, 2023.08.30.555055. https://doi.org/10.1101/2023.08.30.555055.

(17) Gueto-Tettay, C.; Tang, D.; Happonen, L.; Heusel, M.; Khakzad, H.; Malmström, J.; Malmström, L. Multienzyme Deep Learning Models Improve Peptide de Novo Sequencing by Mass Spectrometry Proteomics. *Plos Comput Biol* **2023**, *19* (1), e1010457. https://doi.org/10.1371/journal.pcbi.1010457.

(18) Ambrosetti, F.; Olsen, T. H.; Olimpieri, P. P.; Jiménez-García, B.; Milanetti, E.; Marcatilli, P.; Bonvin, A. M. J. J. ProABC-2: PRediction Of AntiBody Contacts v2 and Its Application to Information-Driven Docking. *Bioinformatics* **2020**, *36* (20), btaa644. https://doi.org/10.1093/bioinformatics/btaa644.

(19) Evans, R.; O'Neill, M.; Pritzel, A.; Antropova, N.; Senior, A.; Green, T.; Žídek, A.; Bates, R.; Blackwell, S.; Yim, J.; Ronneberger, O.; Bodenstein, S.; Zielinski, M.; Bridgland, A.; Potapenko, A.; Cowie, A.; Tunyasuvunakool, K.; Jain, R.; Clancy, E.; Kohli, P.; Jumper, J.; Hassabis, D. Protein Complex Prediction with AlphaFold-Multimer. *Biorxiv* **2022**, 2021.10.04.463034. https://doi.org/10.1101/2021.10.04.463034.

(20) Bryant, P.; Pozzati, G.; Elofsson, A. Improved Prediction of Protein-Protein Interactions Using AlphaFold2. *Nat. Commun.* **2022**, *13* (1), 1265. https://doi.org/10.1038/s41467-022-28865-w.

(21) Kolbowski, L.; Combe, C.; Rappsilber, J. XiSPEC: Web-Based Visualization, Analysis and Sharing of Proteomics Data. *Nucleic Acids Res.* **2018**, *46* (Web Server issue), gky353-. https://doi.org/10.1093/nar/gky353.

(22) Lau, A. M.; Claesen, J.; Hansen, K.; Politis, A. Deuteros 2.0: Peptide-Level Significance Testing of Data from Hydrogen Deuterium Exchange Mass Spectrometry. *Bioinformatics* **2020**, *37* (2), btaa677-. https://doi.org/10.1093/bioinformatics/btaa677.

(23) Lau, A. M. C.; Ahdash, Z.; Martens, C.; Politis, A. Deuteros: Software for Rapid Analysis and Visualization of Data from Differential Hydrogen Deuterium Exchange-Mass Spectrometry. *Bioinformatics* **2019**, *35* (17), btz022-. https://doi.org/10.1093/bioinformatics/btz022.

(24) Olson, R. D.; Assaf, R.; Brettin, T.; Conrad, N.; Cucinell, C.; Davis, J. J.; Dempsey, D. M.; Dickerman, A.; Dietrich, E. M.; Kenyon, R. W.; Kuscuoglu, M.; Lefkowitz, E. J.; Lu, J.; Machi, D.; Macken, C.; Mao, C.; Niewiadomska, A.; Nguyen, M.; Olsen, G. J.; Overbeek, J. C.; Parrello, B.; Parrello, V.; Porter, J. S.; Pusch, G. D.; Shukla, M.; Singh, I.; Stewart, L.; Tan, G.; Thomas, C.; VanOeffelen, M.; Vonstein, V.; Wallace, Z. S.; Warren, A. S.; Wattam, A. R.; Xia, F.; Yoo, H.; Zhang, Y.; Zmasek, C. M.; Scheuermann, R. H.; Stevens, R. L. Introducing the Bacterial and Viral Bioinformatics Resource Center (BV-BRC): A Resource Combining PATRIC, IRD and ViPR. *Nucleic Acids Res.* **2022**, *51* (D1), D678–D689. https://doi.org/10.1093/nar/gkac1003.

(25) Camacho, C.; Coulouris, G.; Avagyan, V.; Ma, N.; Papadopoulos, J.; Bealer, K.; Madden, T. L. BLAST+: Architecture and Applications. *BMC Bioinform.* **2009**, *10* (1), 421. https://doi.org/10.1186/1471-2105-10-421.

(26) Dauparas, J.; Anishchenko, I.; Bennett, N.; Bai, H.; Ragotte, R. J.; Milles, L. F.; Wicky, B. I. M.; Courbet, A.; Haas, R. J. de; Bethel, N.; Leung, P. J. Y.; Huddy, T. F.; Pellock, S.; Tischer, D.; Chan, F.; Koepnick, B.; Nguyen, H.; Kang, A.; Sankaran, B.; Bera, A. K.; King, N. P.; Baker, D. Robust Deep Learning–Based Protein Sequence Design Using ProteinMPNN. *Science* **2022**, *378* (6615), 49–56. https://doi.org/10.1126/science.add2187.

(27) Jumper, J.; Evans, R.; Pritzel, A.; Green, T.; Figurnov, M.; Ronneberger, O.; Tunyasuvunakool, K.; Bates, R.; Žídek, A.; Potapenko, A.; Bridgland, A.; Meyer, C.; Kohl, S. A. A.; Ballard, A. J.; Cowie, A.; Romera-Paredes, B.; Nikolov, S.; Jain, R.; Adler, J.; Back, T.; Petersen, S.; Reiman, D.; Clancy, E.; Zielinski, M.; Steinegger, M.; Pacholska, M.; Berghammer, T.; Bodenstein, S.; Silver, D.; Vinyals, O.; Senior, A. W.; Kavukcuoglu, K.; Kohli, P.; Hassabis, D. Highly Accurate Protein Structure Prediction with AlphaFold. *Nature* **2021**, *596* (7873), 583–589. https://doi.org/10.1038/s41586-021-03819-2.

(28) Pettersen, E. F.; Goddard, T. D.; Huang, C. C.; Couch, G. S.; Greenblatt, D. M.; Meng, E. C.; Ferrin, T. E. UCSF Chimera—A Visualization System for Exploratory Research and Analysis. *J. Comput. Chem.* **2004**, *25* (13), 1605–1612. https://doi.org/10.1002/jcc.20084.

(29) Perez-Riverol, Y.; Bai, J.; Bandla, C.; García-Seisdedos, D.; Hewapathirana, S.; Kamatchinathan, S.; Kundu, D. J.; Prakash, A.; Frericks-Zipper, A.; Eisenacher, M.; Walzer, M.; Wang, S.; Brazma, A.; Vizcaíno, J. A. The PRIDE Database Resources in 2022: A Hub for Mass Spectrometry-Based Proteomics Evidences. *Nucleic Acids Res.* **2021**, *50* (D1), D543–D552. https://doi.org/10.1093/nar/gkab1038.
